# Supplementary material for: Multicolour synthesis in lanthanide-doped nanocrystals through cation exchange in water
Source: Nat Commun. 2016 Oct 4;7:13059. doi: 10.1038/ncomms13059 (PMC5059460; doi:10.1038/ncomms13059)
Supplement: Supplementary Information — Supplementary Figures 1-36, Supplementary Tables 1-2, Supplementary Notes 1-7, Supplementary Methods and Supplementary References. [file ncomms13059-s1.pdf]

Supplementary Figures

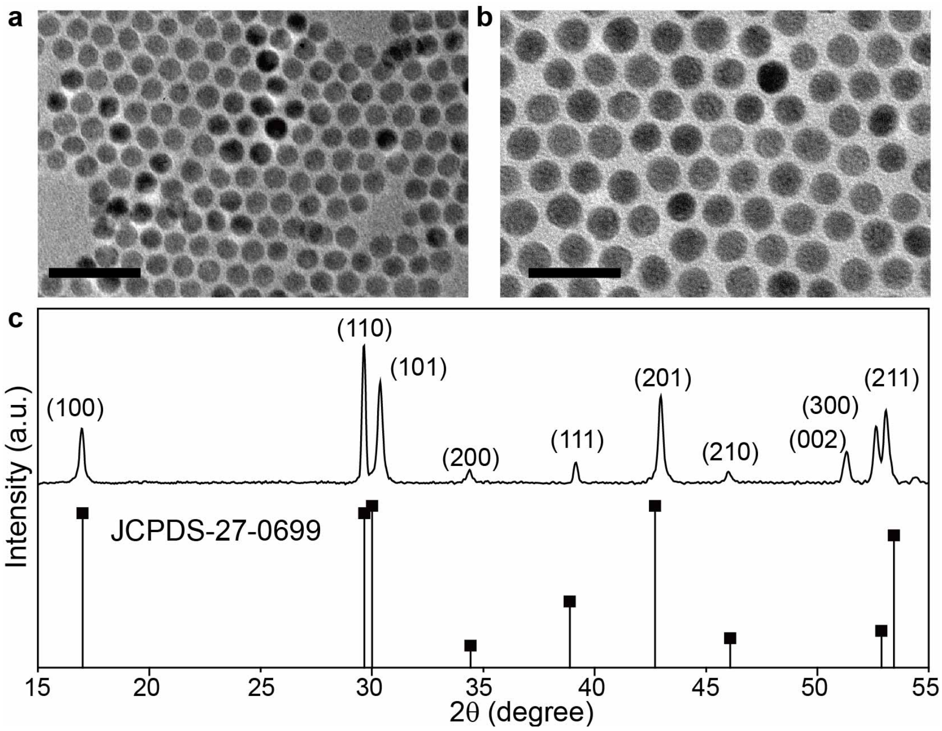

Supplementary Figure 1. Structural characterizations of the as-synthesized $NaGdF_4$:Yb/Tm@$NaGdF_4$ nanocrystals. **a**, TEM imaging of $NaGdF_4$:Yb/Tm (49/1%) core nanocrystals. **b**, TEM imaging of $NaGdF_4$:Yb/Tm@$NaGdF_4$ core-shell nanocrystals. Scale bars are 50 nm. **c**, X-ray powder diffraction pattern of the as-prepared core-shell nanocrystals showing that all peaks can be well indexed in accordance with hexagonal-phase $NaGdF_4$ structure (Joint Committee on Powder Diffraction Standards file number 27-0699).

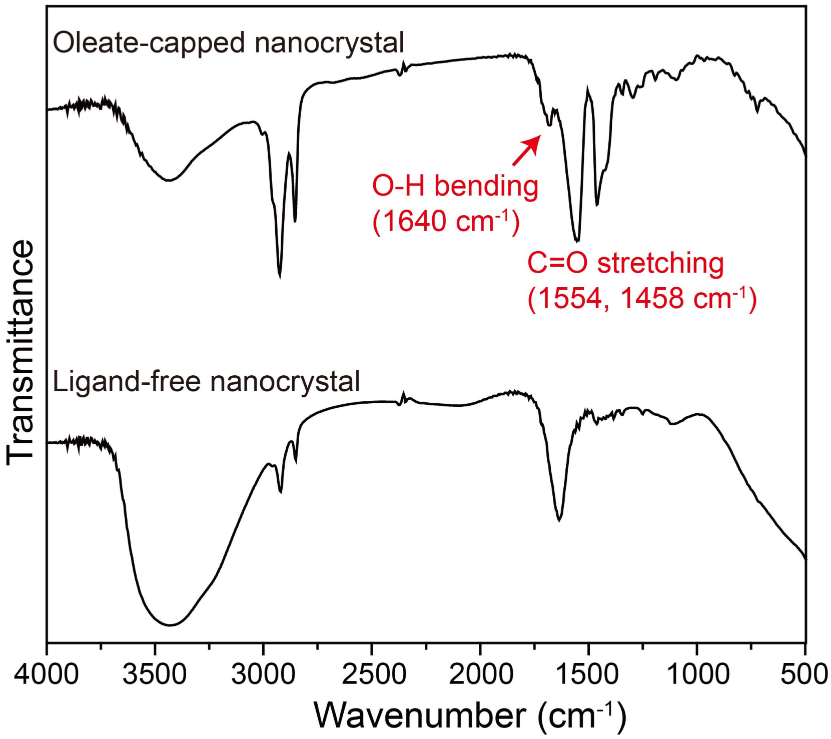

Supplementary Figure 2. Fourier transform infrared spectroscopy (FTIR) of the as-prepared oleate-capped (top) and ligand-free (bottom) $NaGdF_4$:Yb/Tm@$NaGdF_4$ core-shell nanocrystals.

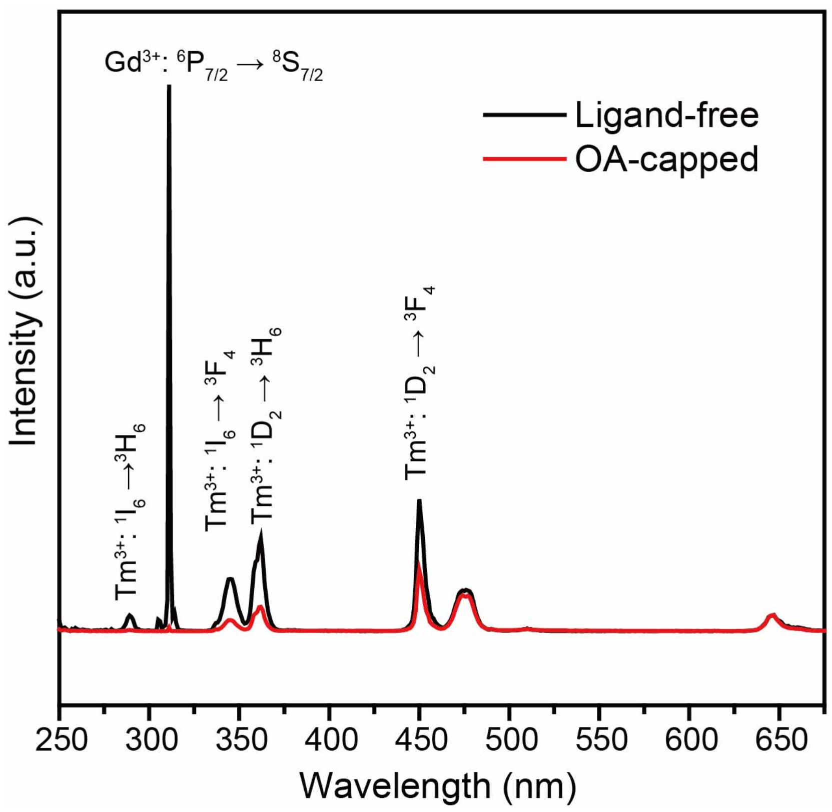

Supplementary Figure 3. Room-temperature photoluminescence of the as-prepared oleate-capped (red line) and ligand-free (black line) $NaGdF_4$:Yb/Tm@$NaGdF_4$ core-shell nanoparticles measured under 980 nm excitation.

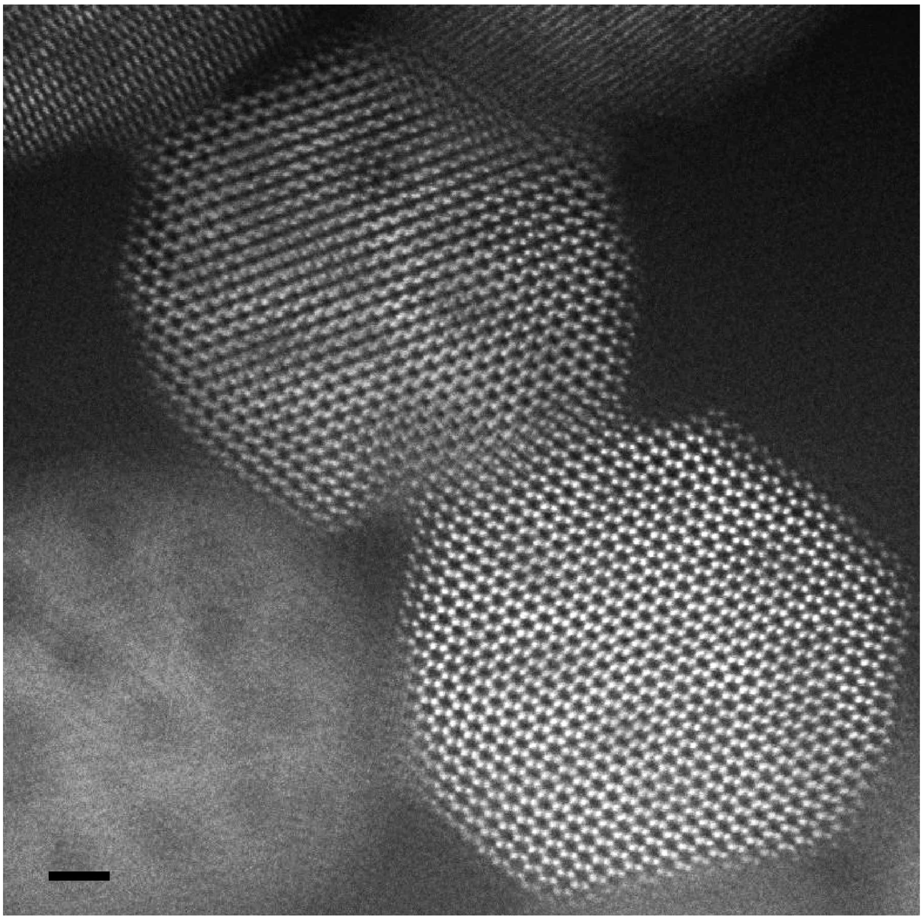

Supplementary Figure 4. The atomic-resolution TEM image of the as-prepared ligand-free $NaGdF_4$:Yb/Tm(49/1%)@$NaGdF_4$ core-shell nanocrystals after cation exchange with Eu^{3+} ions, suggesting the preservation of the high crystalline nature of the nanocrystals. Scale bar: 2 nm.

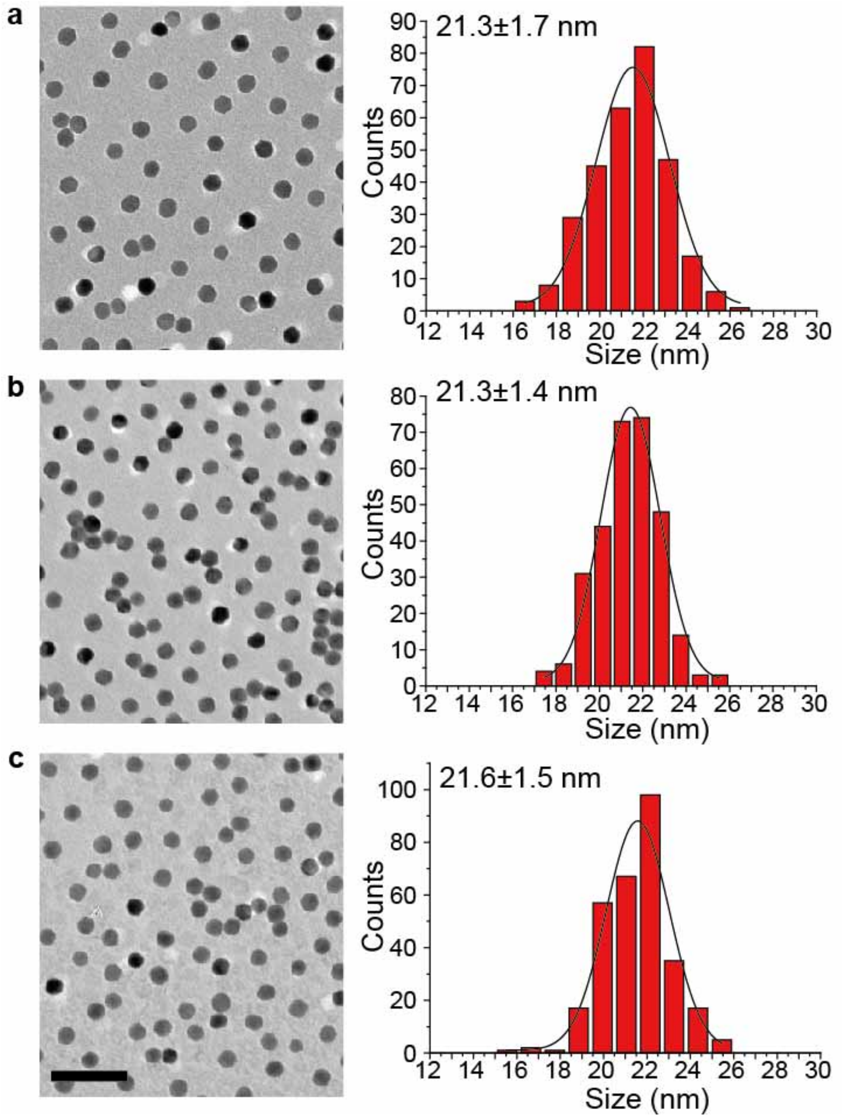

Supplementary Figure 5. Size and morphology investigation of the nanocrystals used for cation exchange at room temperature. Low-resolution TEM images (left panel) and the corresponding size histograms (right panel) of $NaGdF_4$:Yb/Tm@$NaGdF_4$ nanocrystals before (**a**) and after cation exchange with Tb^{3+} (**b**) and Eu^{3+} (**c**) ions at room temperature. Scale bar: 100 nm.

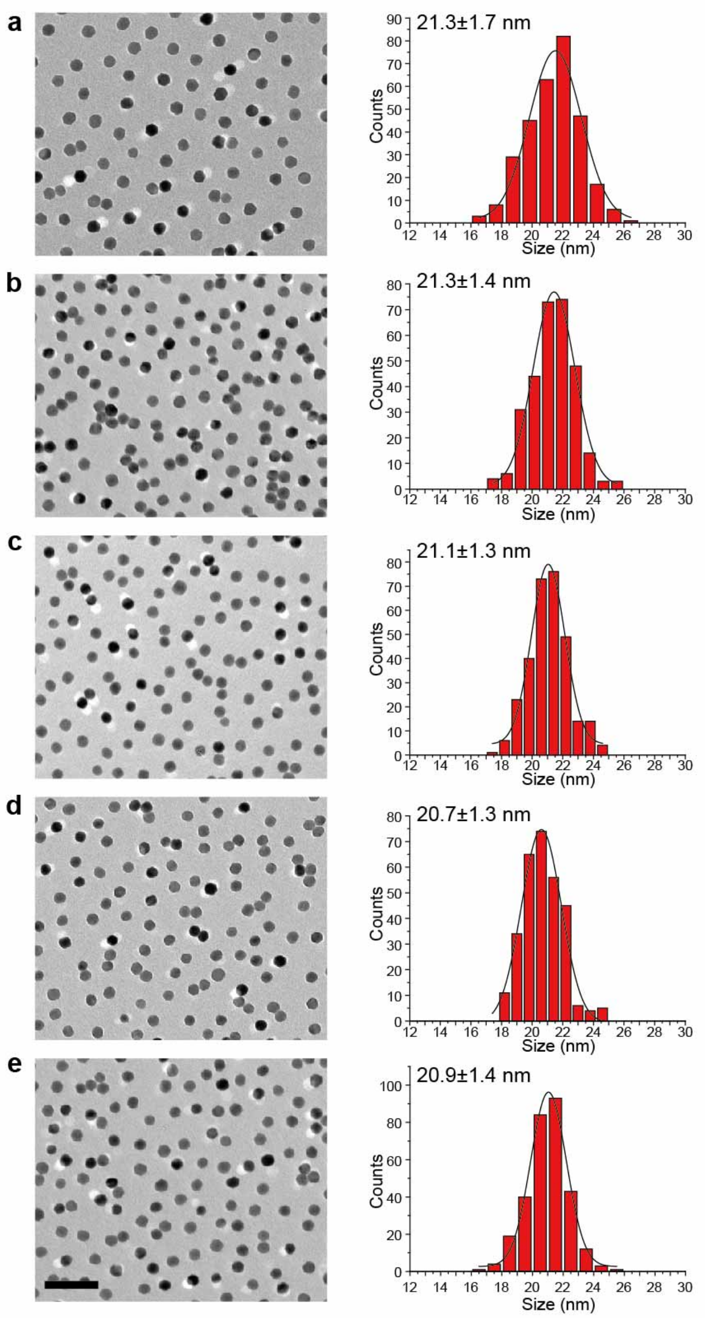

Supplementary Figure 6. Size and morphology investigation of the nanocrystals used for cation exchange with Tb^{3+} at varying temperatures. Low-resolution TEM images (left panel) and the corresponding size histograms (right panel) of $NaGdF_4$:Yb/Tm@$NaGdF_4$ nanocrystals before (**a**) and after cation exchange with Tb^{3+} ions at 25 (**b**), 50 (**c**), 70 (**d**), and 90 °C (**e**). Scale bar: 100 nm.

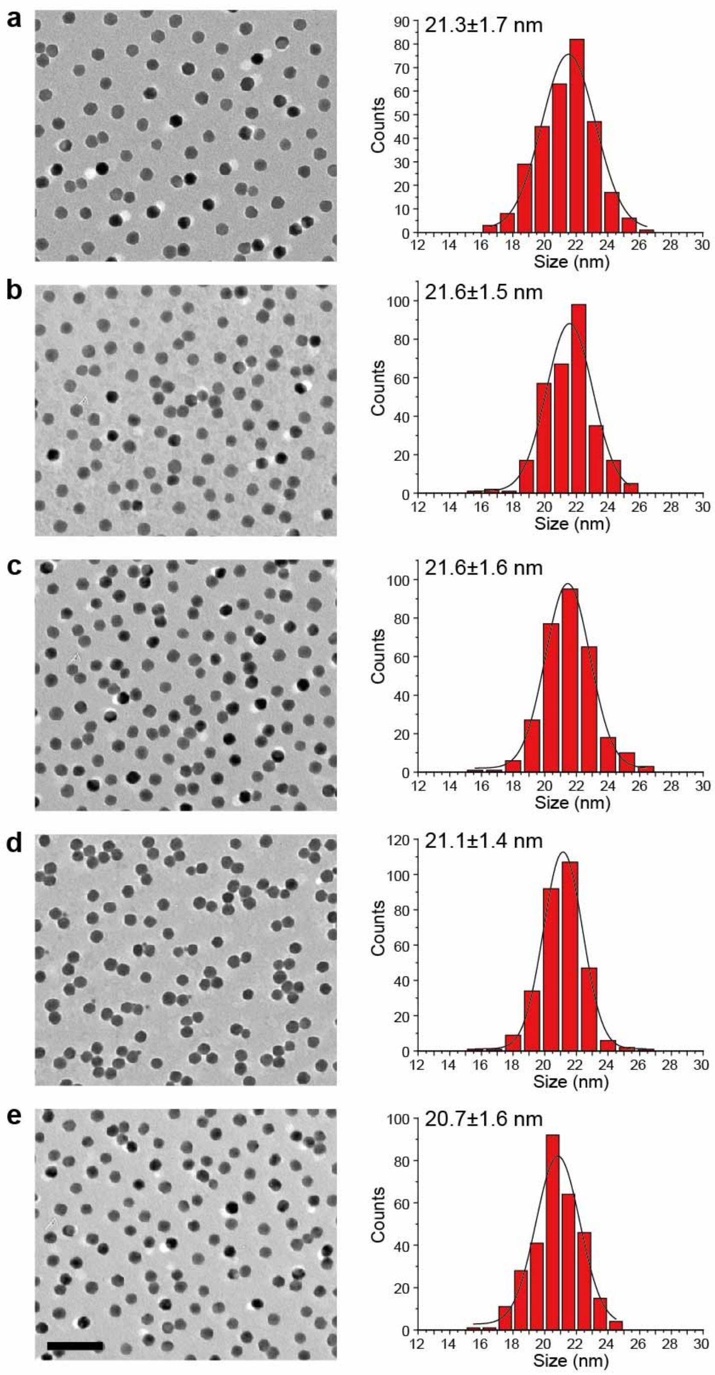

Supplementary Figure 7. Size and morphology investigation of the nanocrystals used for cation exchange with Eu^{3+} at varying temperatures. Low-resolution TEM images (left panel) and the corresponding size histograms (right panel) of $NaGdF_4$:Yb/Tm@$NaGdF_4$ nanocrystals before (**a**) and after cation exchange with Eu^{3+} ions at 25 (**b**), 50 (**c**), 70 (**d**), and 90 ^{o}C (**e**). Scale bar: 100 nm.

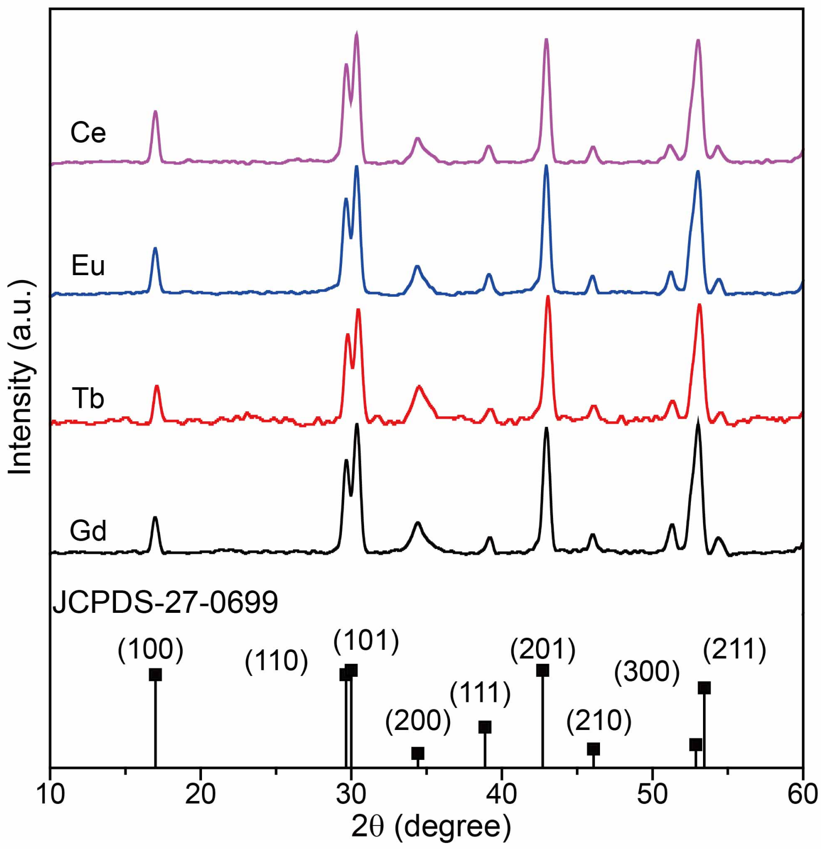

Supplementary Figure 8. Phase investigation of the nanocrystals after cation exchange at room temperature. X-ray powder diffraction patterns of $NaGdF_4$:Yb/Tm@$NaGdF_4$ nanocrystals collected before (black line) and after cation exchange with Tb^{3+} (red line), Eu^{3+} (blue line) and Ce^{3+} (purple line) ions, respectively.

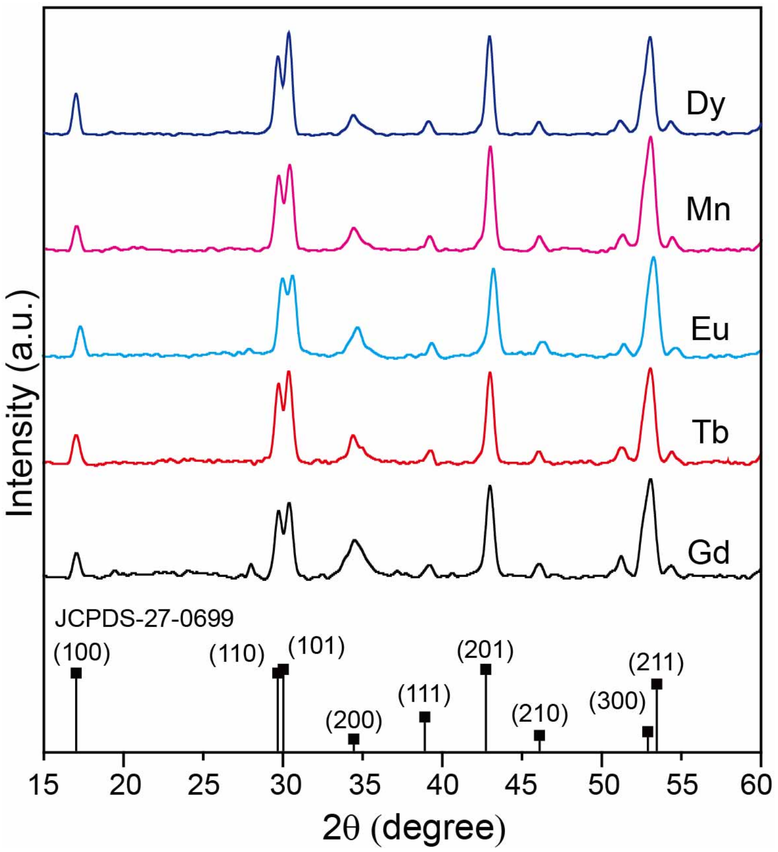

Supplementary Figure 9. Phase investigation of the nanocrystals after cation exchange at 90 °C. X-ray powder diffraction patterns of $NaGdF_4$:Yb/Tm@$NaGdF_4$ nanocrystals obtained before (black line) and after cation exchange with Tb^{3+}, Eu^{3+}, Dy^{3+} and Mn^{2+} ions, respectively.

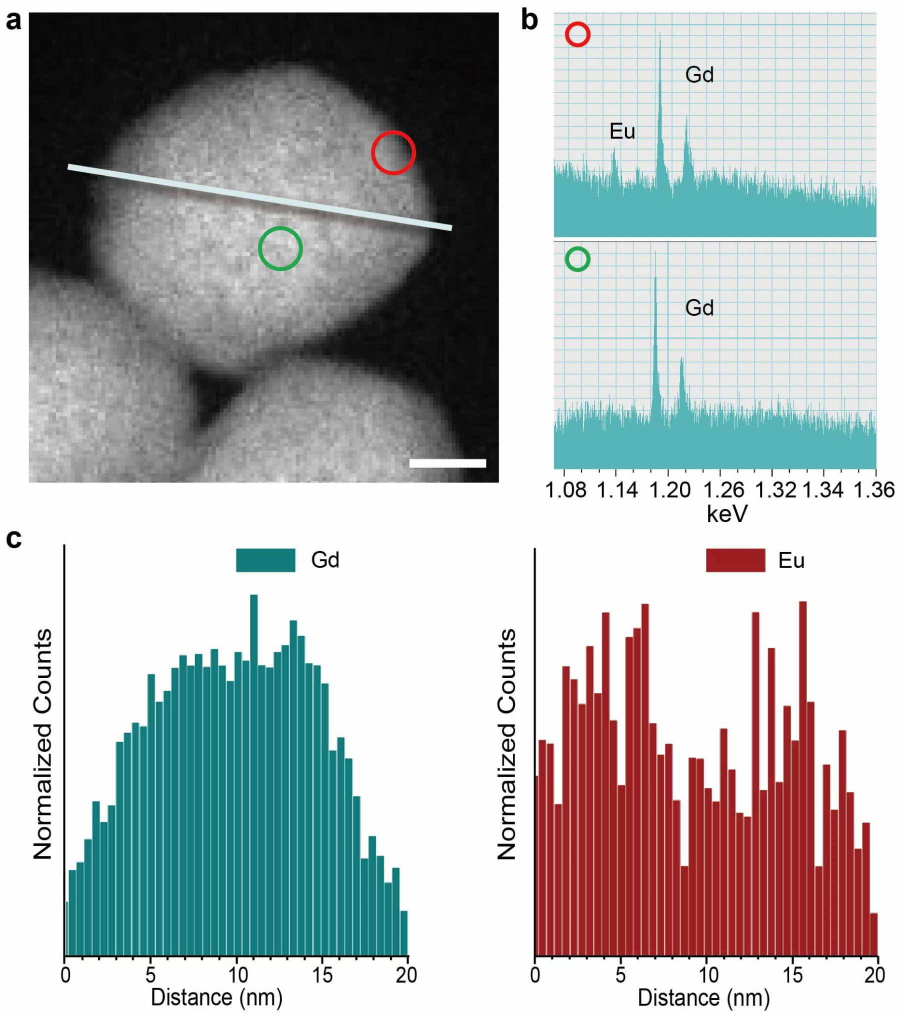

Supplementary Figure 10. The elemental analysis of Eu^{3+}-exchanged nanocrystals. a, STEM image of the as-prepared ligand-free $NaGdF_4$:Yb/Tm@$NaGdF_4$ nanoparticles obtained after cation exchange with Eu^{3+} ions. Scale bar: 5 nm. **b**, The corresponding electron energy loss spectroscopy (EELS) point analysis conducted with STEM imaging on areas located in the center (green circle) and at the edge (red circle) of a nanocrystal, respectively. **c**, The corresponding elemental mapping of Gd^{3+} (left) and Eu^{3+} (right) by STEM line scanning of the single nanocrystal. The combined EELS point analysis and line scan results clearly show a higher Gd content in the inner layer and a higher Eu content in the shell layer, which can be ascribed to the partial substitution of Gd^{3+} by Eu^{3+} at the particle surface during the cation-exchange process.

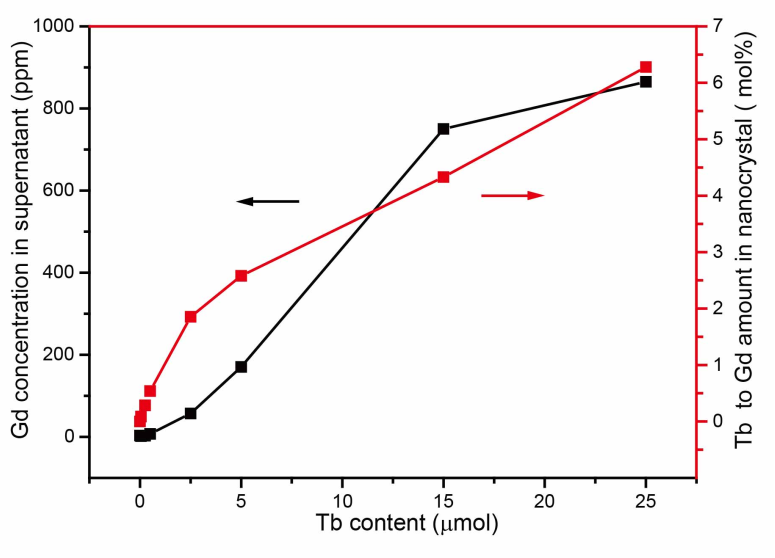

Supplementary Figure 11. Inductively coupled plasma mass spectroscopic (ICP-MS) analysis. The black line denotes the amount of Gd^{3+} ions released from the $NaGdF_4$:Yb/Tm@$NaGdF_4$ nanocrystals upon cation exchange with increasing Tb^{3+} concentration. The red line corresponds to relative Tb^{3+}-to-Gd^{3+} content in the nanocrystals upon cation exchange with increasing Tb^{3+}concentration.

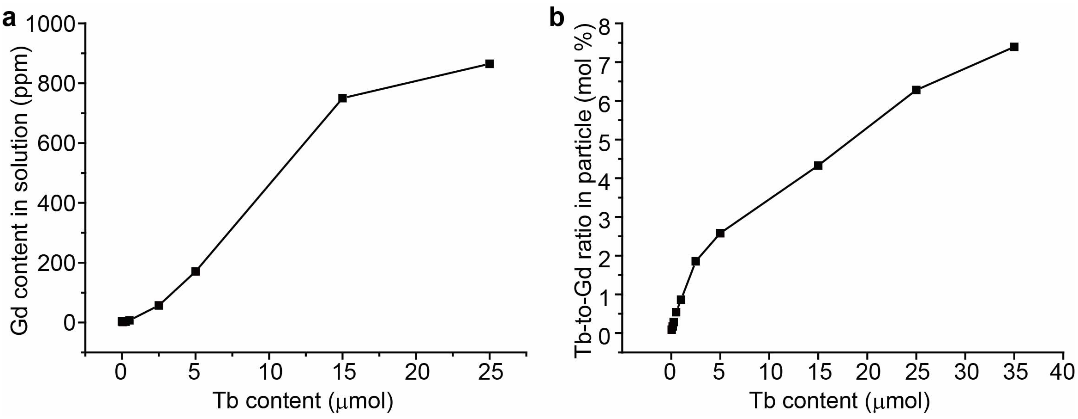

Supplementary Figure 12. Inductively coupled plasma mass spectroscopic (ICP-MS) analysis. a, Plot of Gd^{3+} amount released from the $NaGdF_4$:Yb/Tm@$NaGdF_4$ nanocrystals upon cation exchange with increasing Tb^{3+} concentration. **b**, Plot of Tb^{3+}/Gd^{3+} molar ratio in the nanocrystals upon cation exchange with increasing Tb^{3+} concentration.

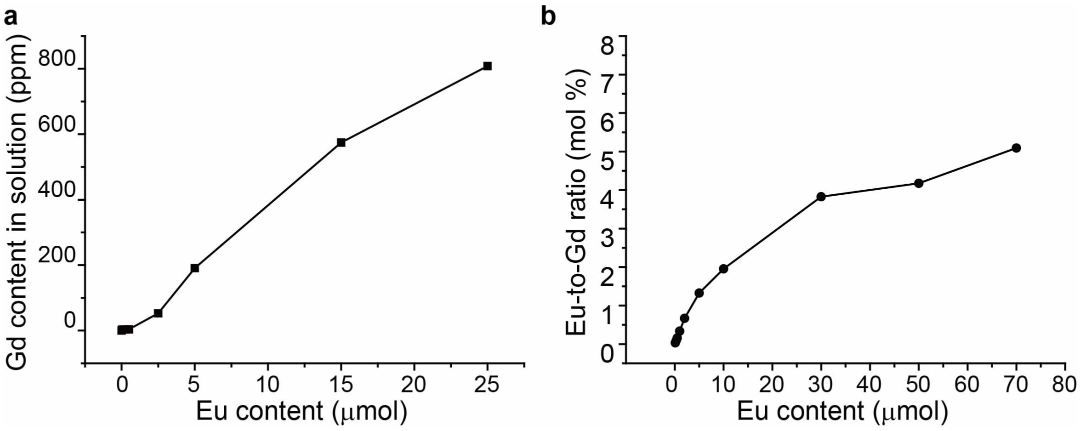

Supplementary Figure 13. Inductively coupled plasma mass spectroscopic (ICP-MS) analysis. a, Plot of Gd^{3+} amount released from the $NaGdF_4$:Yb/Tm@$NaGdF_4$ nanocrystals upon cation exchange with increasing Eu^{3+} concentration. **b**, Plot of Eu^{3+}/Gd^{3+} molar ratio in the nanocrystals upon cation exchange with increasing Eu^{3+} concentration.

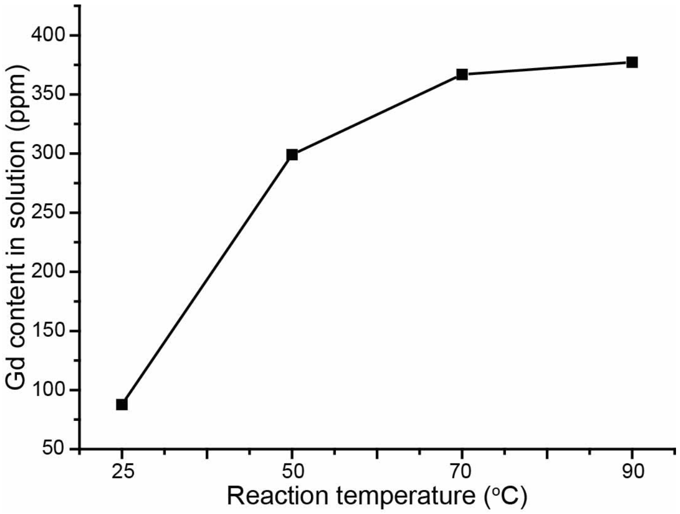

Supplementary Figure 14. ICP-MS analysis of Gd^{3+} content released from the $NaGdF_4$:Yb/Tm@$NaGdF_4$ nanocrystals after cation exchange with $TbCl_3$ as a function of reaction temperature.

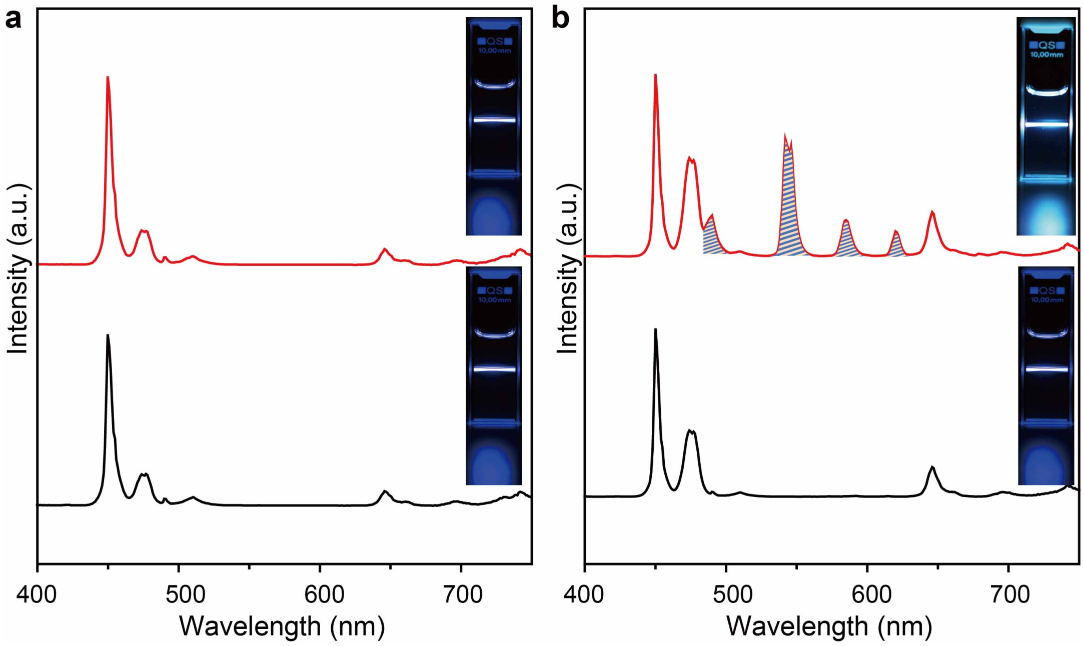

Supplementary Figure 15. Comparative emission spectrum analysis confirming the important role of energy migration in color manipulation involving core-shell nanocrystals. a, Upconversion luminescence spectra of ligand-free $NaYF_4$:Yb/Tm(30/0.5%)@$NaYF_4$ nanocrystals before (black line) and after (red line) treatment with Tb^{3+} ions. **b**, Upconversion luminescence spectra of ligand-free $NaGdF_4$:Yb/Tm(49/1%)@$NaGdF_4$ nanocrystals before (black line) and after (red line) treatment with Tb^{3+} ions. The insets are the corresponding luminescence photographs of the sample solutions under irradiation with a 980 nm laser.

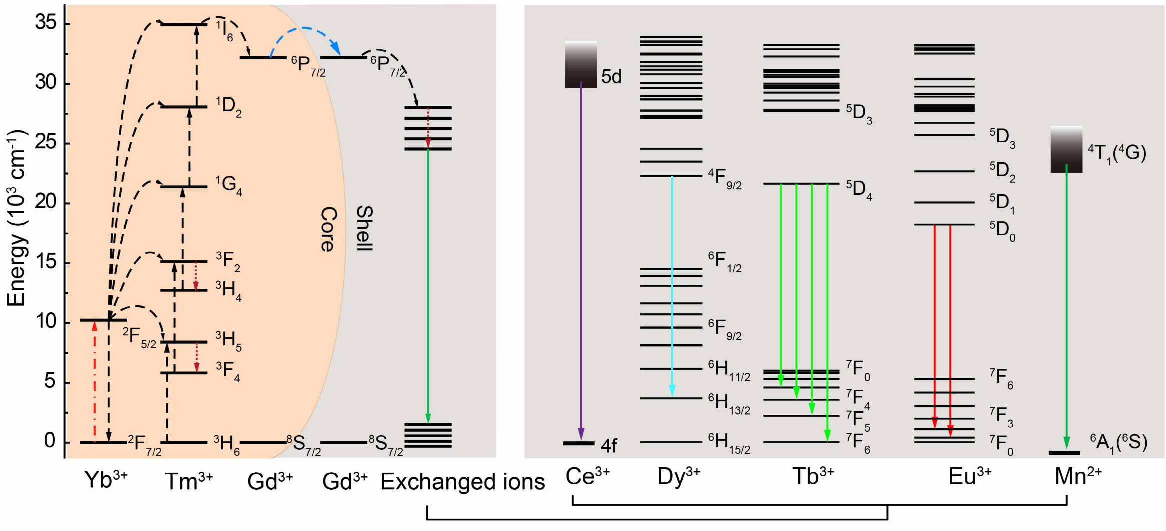

Supplementary Figure 16. Proposed energy transfer mechanisms of the cation exchange-induced color tuning in the $NaGdF_4$:Yb/Tm@$NaGdF_4$ nanocrystals. Note that only partial energy levels of Tm^{3+}, Gd^{3+} and exchanged ions (Ce^{3+}, Dy^{3+}, Tb^{3+}, Eu^{3+} and Mn^{2+}) are shown for clarity. The dashed/dotted, dashed, dotted, and full arrows represent photon excitation, energy transfer, cross-relaxation, and emission processes, respectively.

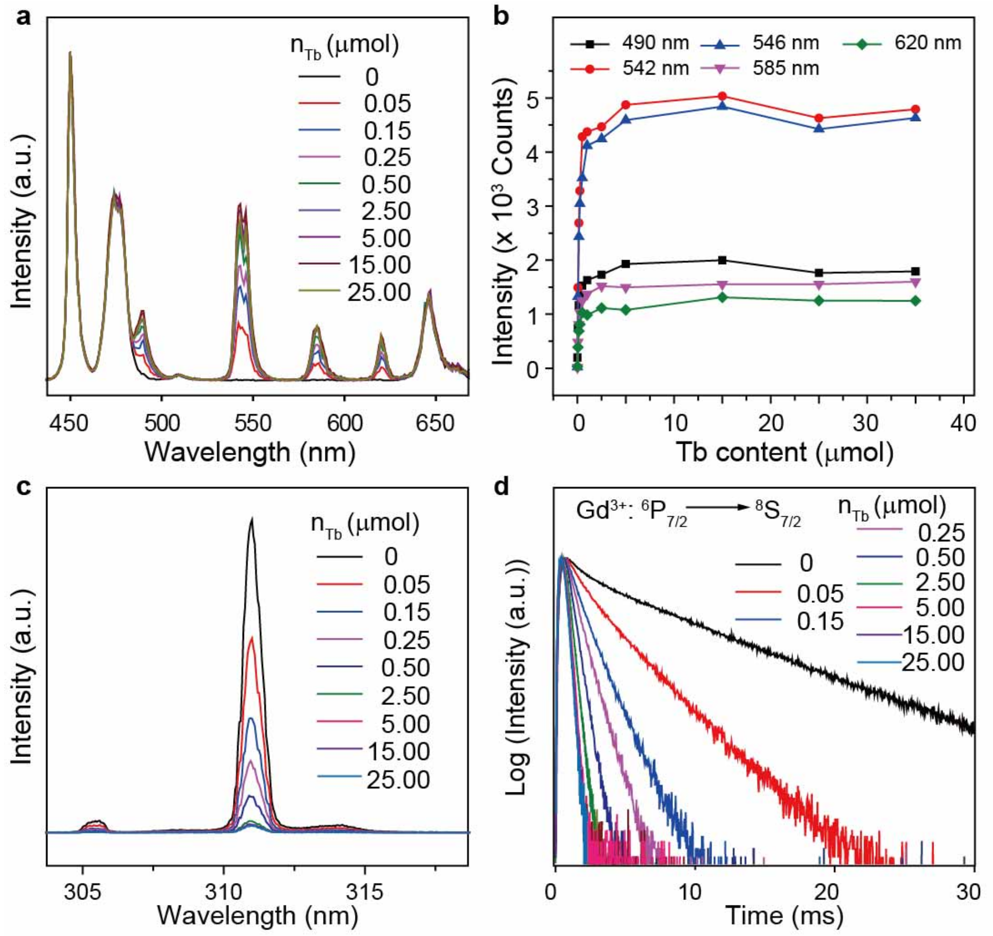

Supplementary Figure 17. Tb^{3+} concentration-dependent emission of the cation-exchanged nanocrystals prepared at room temperature. **a**, Upconversion emission spectra of $NaGdF_4$:Yb/Tm@$NaGdF_4$ nanoparticles plotted as a function of Tb^{3+} concentration under excitation at 980 nm. **b**, The intensity change in Tb^{3+} emission at different wavelengths plotted as a function of concentration. **c**, The intensity change in Gd^{3+} emission at 311 nm, showing a decreasing trend as a function of Tb^{3+} concentration. **d**, Photoluminescence decay curves of Gd^{3+} at 311 nm with varied Tb^{3+} concentrations.

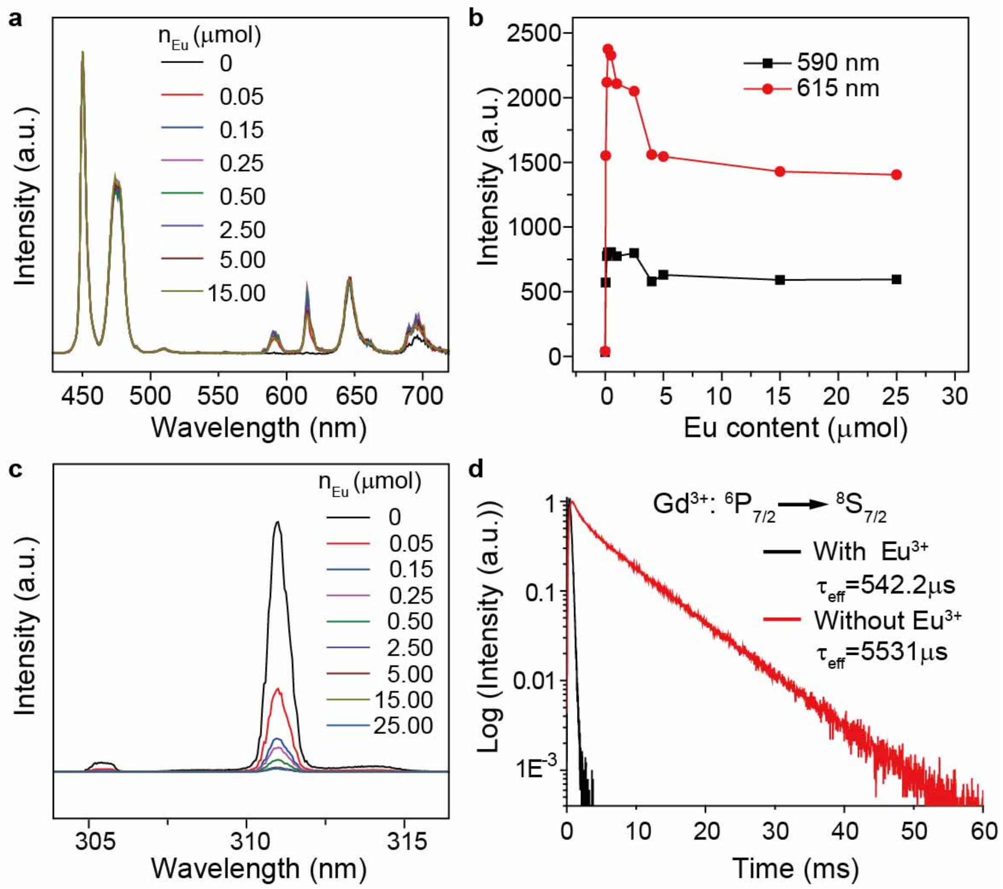

Supplementary Figure 18. Eu^{3+} concentration-dependent emission of the cation-exchanged nanocrystals prepared at room temperature. **a**, Upconversion emission spectra of $NaGdF_4$:Yb/Tm@$NaGdF_4$ nanoparticles plotted against Eu^{3+} concentration under excitation at 980 nm. **b**, The intensity change in Eu^{3+} emission at different wavelengths as a function of concentration. **c**, The intensity change in Gd^{3+} emission at 311 nm as a function of Eu^{3+} concentration. **d**, Photoluminescence decay curves of Gd^{3+} at 311 nm measured with and without the Eu^{3+} exchange process.

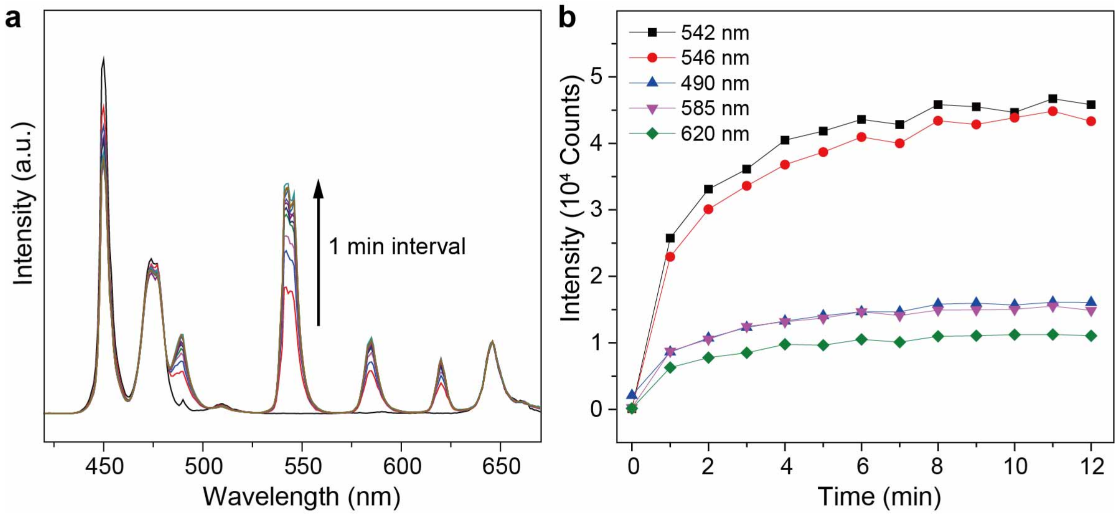

Supplementary Figure 19. Time-dependent photoluminescence of the nanocrystals after cation exchange. a, Upconversion emission spectra of nanoparticles (1 mL), prepared by mixing $NaGdF_4$:Yb/Tm@$NaGdF_4$ nanocrystals (26.2 mg) with $TbCl_3$ (20 μmol), as a function of cation exchange time under excitation at 980 nm with a CW diode laser. **b**, The corresponding intensity change in Tb^{3+} emission of different wavelength plotted against the reaction time.

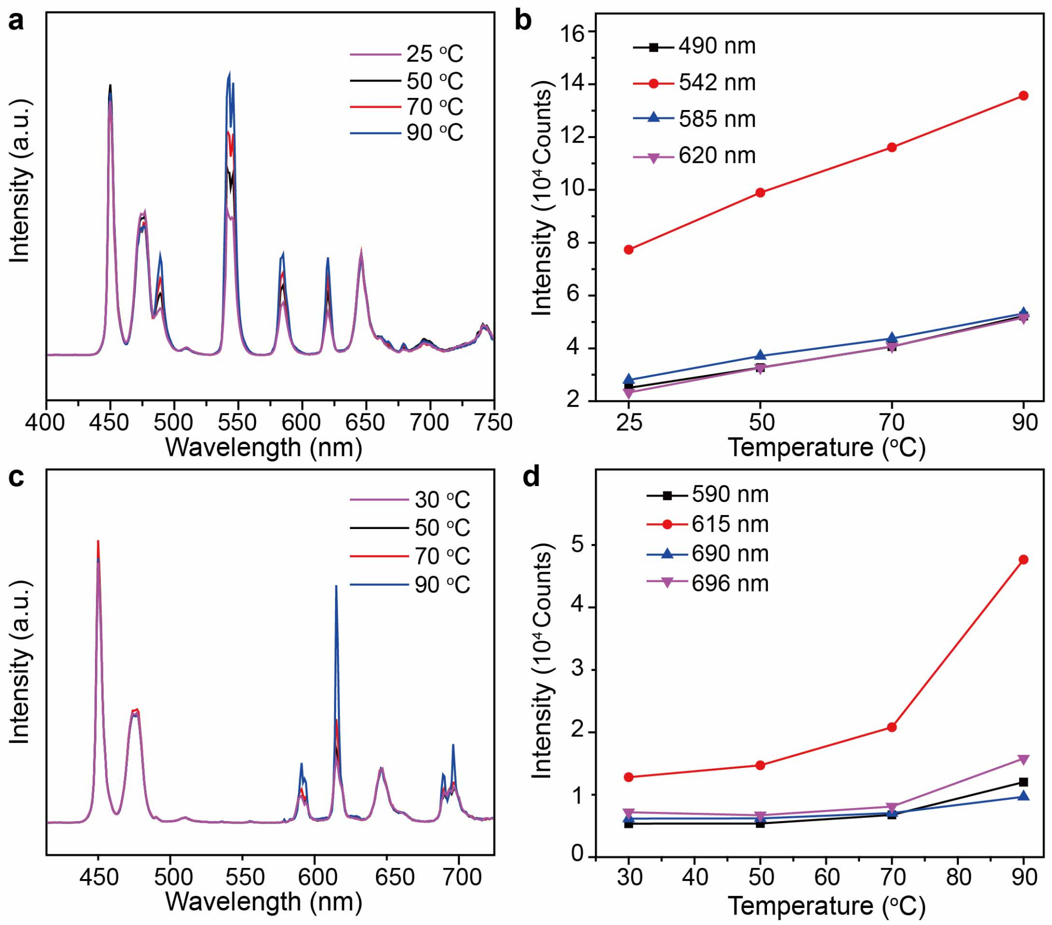

Supplementary Figure 20. Temperature-dependent emission profiles of the cation-exchanged nanocrystals. a, Upconversion emission spectra of the $NaGdF_4$:Yb/Tm@$NaGdF_4$ nanocrystals obtained after cation exchange with Tb^{3+} at varying temperatures. **b**, The intensity change in Tb^{3+} emission as a function of reaction temperature. **c**, Upconversion emission spectra of $NaGdF_4$:Yb/Tm@$NaGdF_4$ nanocrystals obtained after treatment with Eu^{3+} at varying temperatures. **d**, The intensity change in Eu^{3+} emission as a function of reaction temperature.

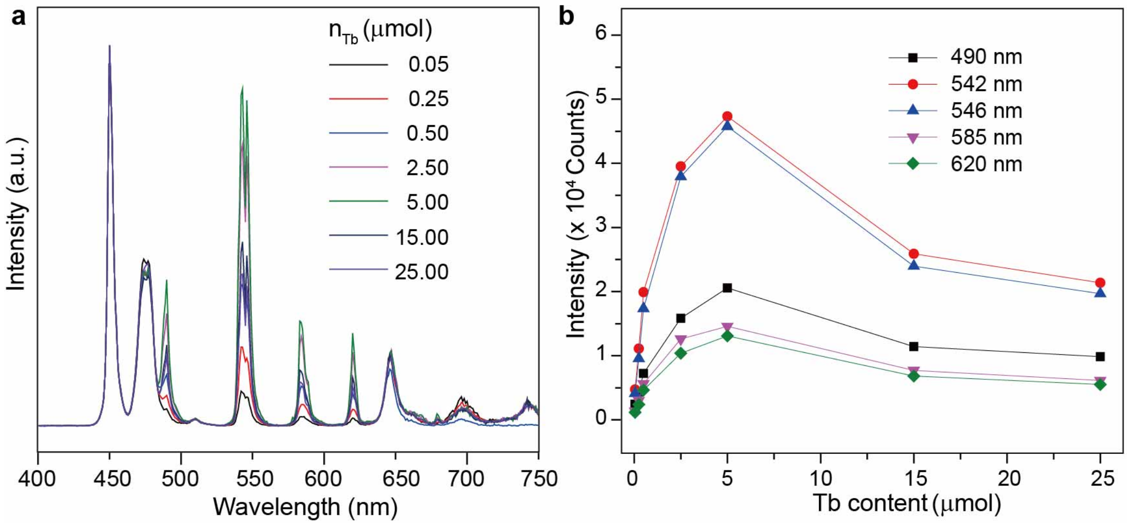

Supplementary Figure 21. Upconversion luminescence of Tb^{3+}-exchanged nanocrystals prepared at 90 °C. a, Upconversion luminescence of the nanocrystals prepared by cation exchange of $NaGdF_4$:Yb/Tm@$NaGdF_4$ nanoparticles with Tb^{3+} of different concentrations at 90 °C. **b**, The corresponding intensity change in Tb^{3+} emission as a function of concentration.

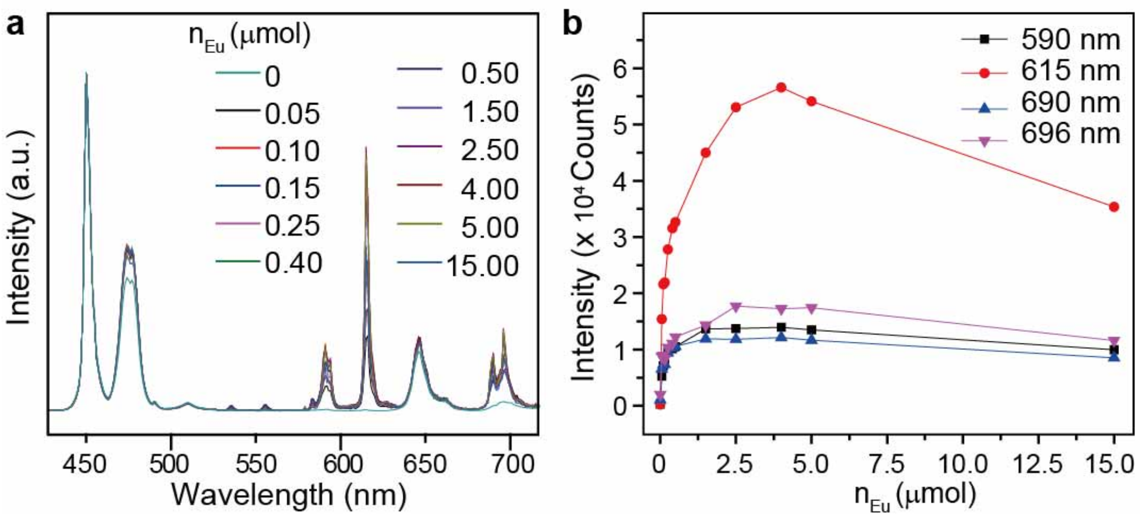

Supplementary Figure 22. Upconversion luminescence of Eu^{3+}-exchanged nanocrystals prepared at 90 °C. a, Upconversion luminescence of the nanocrystals prepared by cation exchange of $NaGdF_4$:Yb/Tm@$NaGdF_4$ nanoparticles with Eu^{3+} of different concentrations at 90 °C. **b**, The corresponding intensity change in Eu^{3+} emission as a function of concentration.

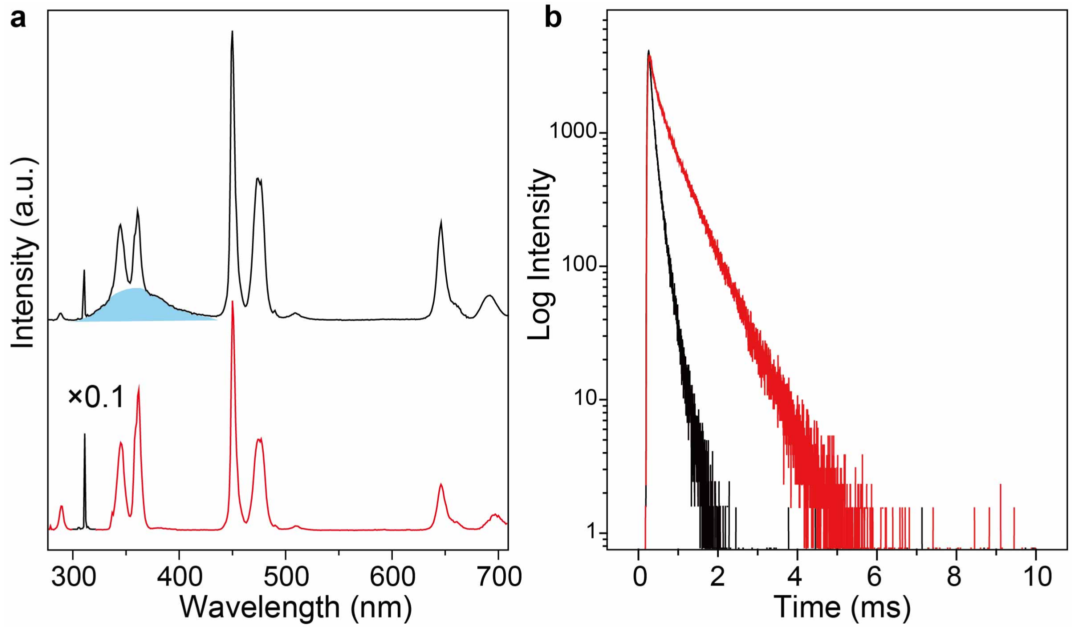

Supplementary Figure 23. Upconversion luminescence of Ce^{3+}-exchanged nanocrystals. **a**, Upconversion emission spectra of the as-prepared $NaGdF_4$:Yb/Tm@$NaGdF_4$ nanoparticles before (red line) and after (black line) treatment with Ce^{3+} (24 μmol). Note that the Ce^{3+} emission is highlighted in cyan color. **b**, Lifetime decay profiles of Gd^{3+} emission at 311 nm from the $NaGdF_4$:Yb/Tm@$NaGdF_4$ nanoparticle before (red line) and after (black line) treatment with Ce^{3+} ions.

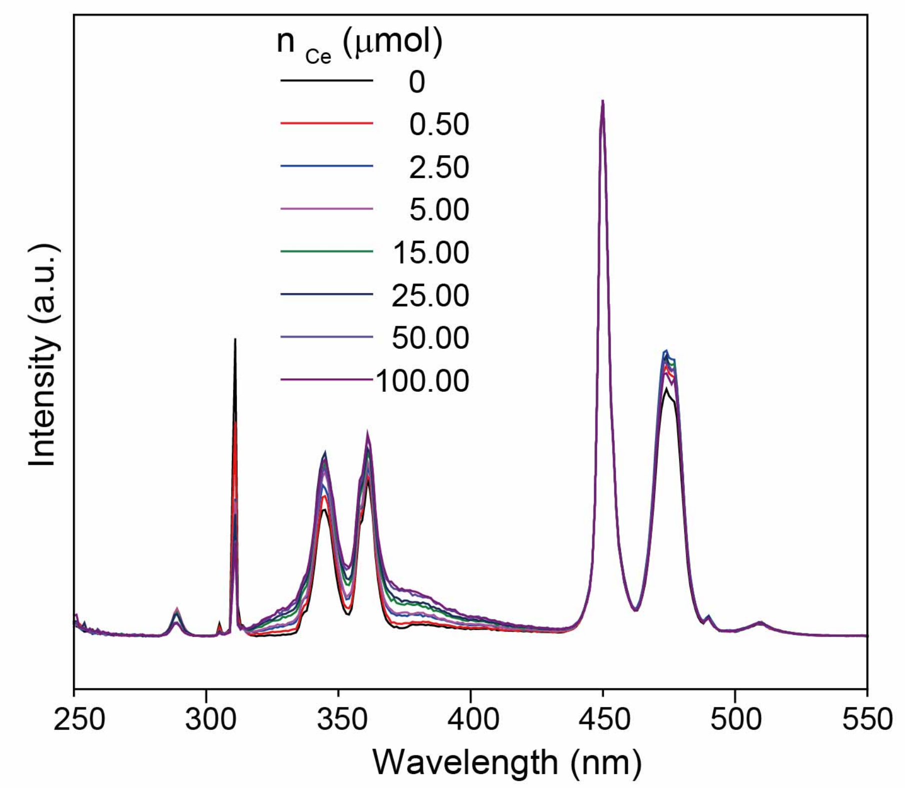

Supplementary Figure 24. Upconversion luminescence of the nanocrystals obtained by treatment of $NaGdF_4$:Yb/Tm@$NaGdF_4$ with Ce^{3+} ions at different concentrations under room temperature.

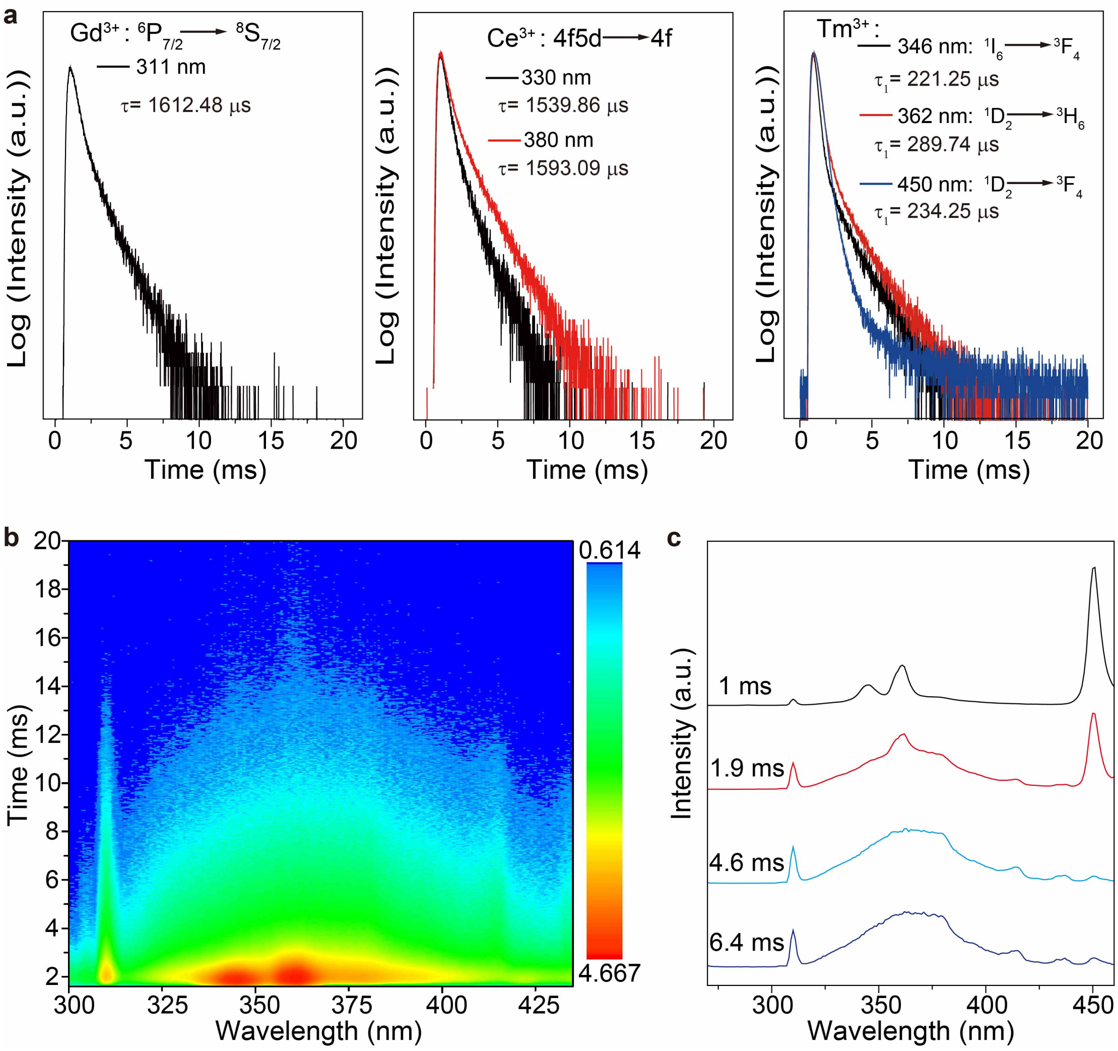

Supplementary Figure 25. Lifetime investigations of Ce^{3+}-exchanged $NaGdF_4$:Yb/Tm@$NaGdF_4$ nanocrystals. a, Decay curve of emissions at 311, 330, 380, 346, 362 and 450 nm, respectively. **b**, Time-resolved emission spectra. The color bar represents the intensity count. **c**, Normalized photoluminescence spectra collected at different decay time intervals (1, 1.9, 4.6, and 6.4 ms) following a pulse excitation at 980nm.

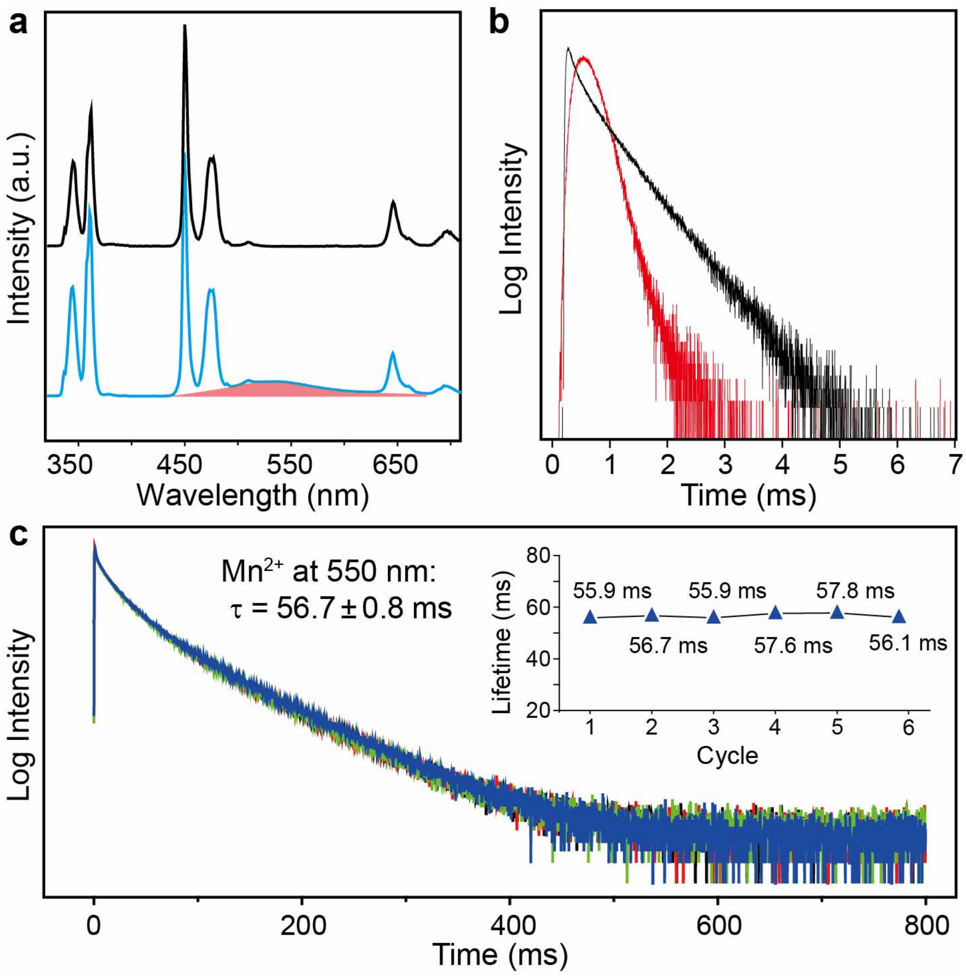

Supplementary Figure 26. Upconversion luminescence of Mn^{2+}-exchanged nanocrystals. a, Upconversion emission of the $NaGdF_4$:Yb/Tm@$NaGdF_4$ nanoparticles before (black line) and after (Cyan line) treatment with Mn^{2+} ions (24 μmol) at 90 °C. Note that the Mn^{2+} emission is highlighted in red color. **b**, The corresponding lifetime decay curves of Gd^{3+} emission at 311 nm recorded from the nanoparticles with (red line) and without (black line) the treatment of cation exchange. **c**, Lifetime decay profiles of Mn^{2+} emission at 550 nm from the nanoparticles after treatment with Mn^{2+} ions. Inset shows the repeatability of the lifetime measurements of Mn^{2+} emission at 550 nm.

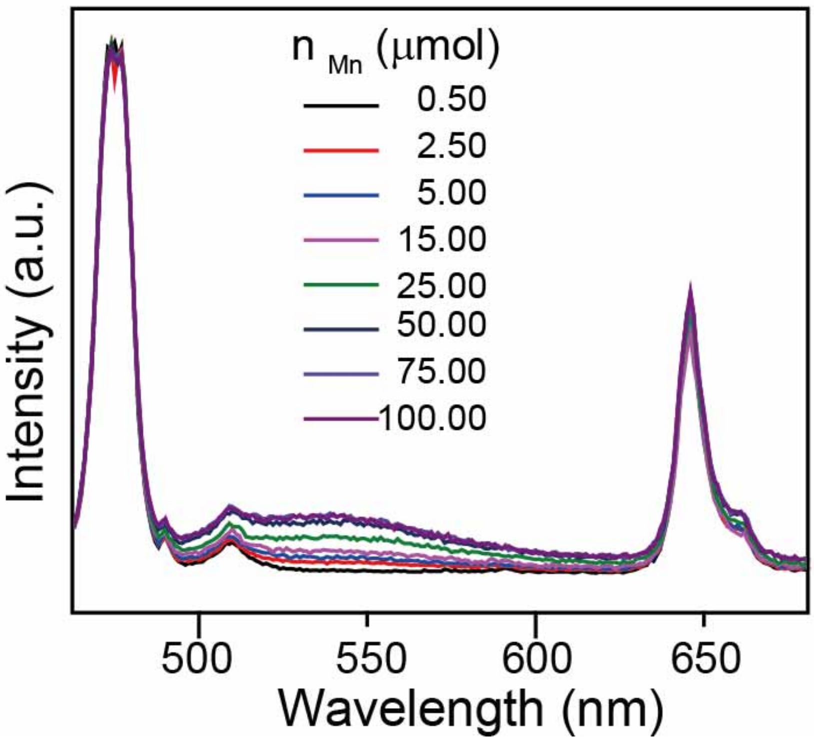

Supplementary Figure 27. Upconversion luminescence of the nanocrystals prepared by cation exchange of $NaGdF_4$:Yb/Tm@$NaGdF_4$ with varied concentrations of Mn^{2+} at 90 °C.

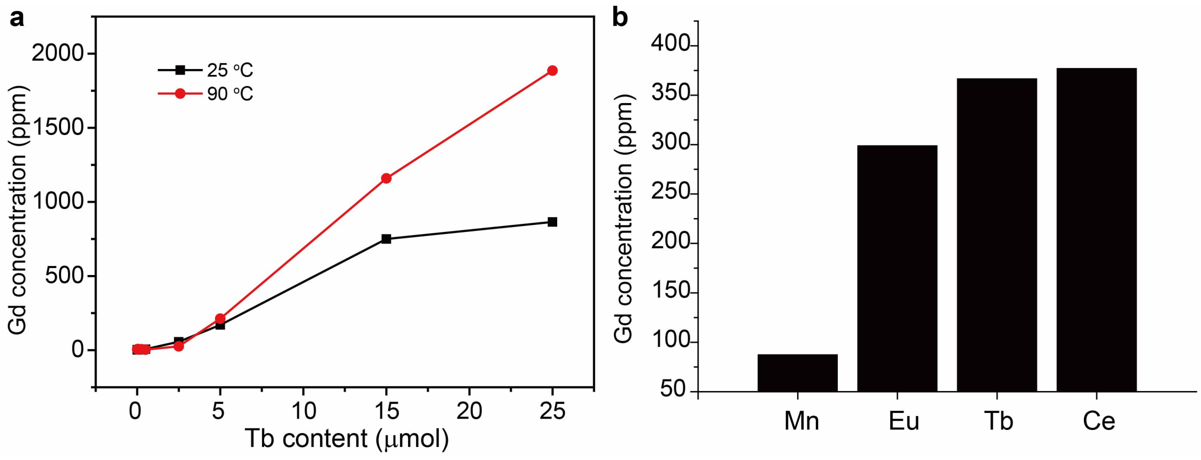

Supplementary Figure 28. Inductively coupled plasma mass spectroscopy (ICP-MS) analysis of Gd content discharged from the nanocrystals after cation exchange. a, The Gd content discharged from the nanocrystals as a function of Tb^{3+} content used for cation exchange with $NaGdF_4$:Yb/Tm@$NaGdF_4$ nanocrystals at 25 °C (black curve) and 90 °C (red curve), respectively. **b,** The Gd concentrations measured in the dialyzed solution of $NaGdF_4$:Yb/Tm@$NaGdF_4$ nanocrystals after cation exchange at 25 °C with various activators (Mn^{2+}, Eu^{3+}, Tb^{3+}, and Ce^{3+}; 5 μmol each).

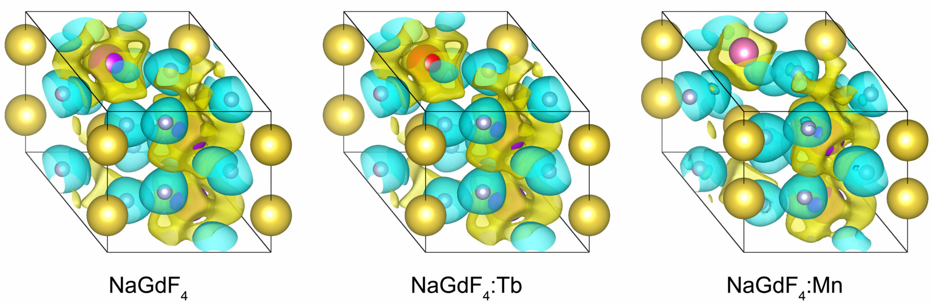

Supplementary Figure 29. Iso-surface of charge transfer in pure, Tb- and Mn-doped β-$NaGdF_4$ nanocrystals. Positive and negative charges are shown in yellow and blue, respectively. It is obvious that the charge transfer mainly occurs between Gd/dopant and F atoms. In the Tb-doped nanocrystal, the charge transfer from Tb to F atoms is similar to that of the pure $NaGdF_4$ nanocrystal, which is consistent with the small formation energy calculated for the Tb-doped $NaGdF_4$ nanocrystal. However, the amount of the charge transferred between Mn and F atoms is much less than obtained between Gd and F (Supplementary Table 2), leading to a decrease in dipole polarizability of the Gd-based lattice. This is in line with the larger formation energies of Mn-doped $NaGdF_4$ nanocrystals as compared to the Tb-doped $NaGdF_4$ nanocrystals. Na atoms: yellow; Gd atoms: purple; F atoms: white; Tb atom: red; and Mn atom: pink.

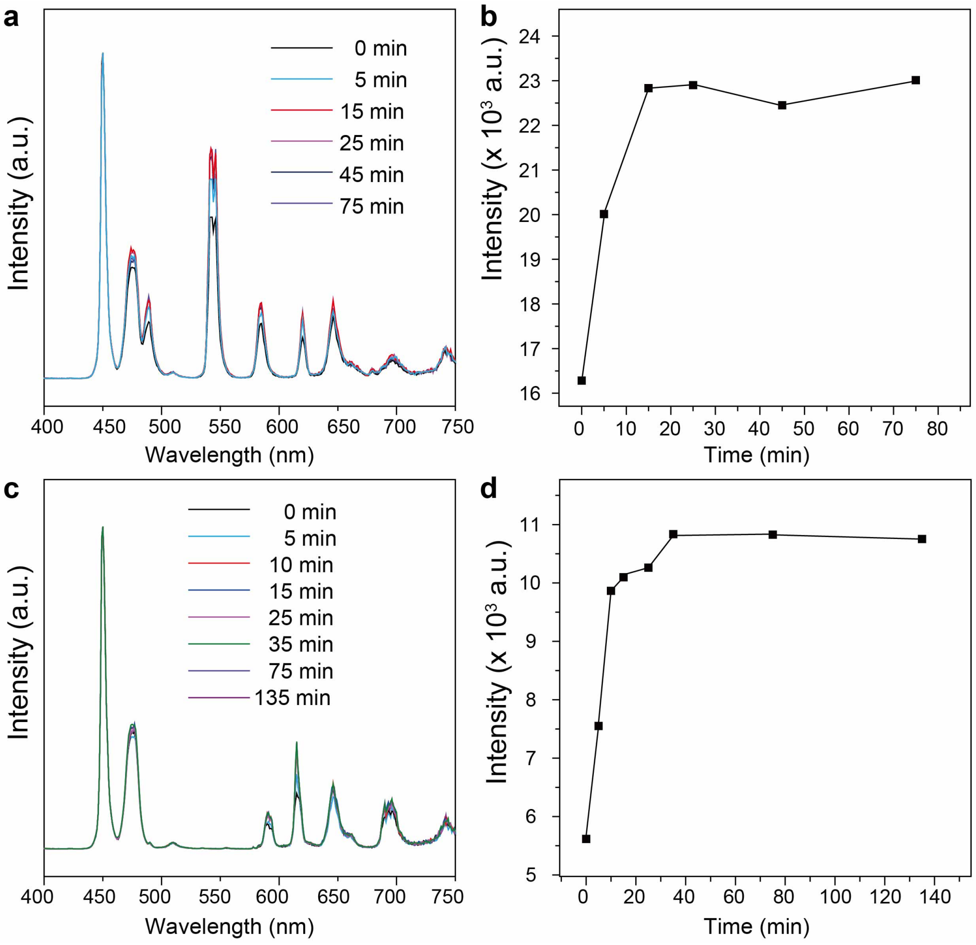

Supplementary Figure 30. Ion diffusion effect on the optical property of the as-prepared exchanged nanocrystals. **a**, Upconversion luminescence of the Tb^{3+}-exchanged $NaGdF_4$:Yb/Tm@$NaGdF_4$ nanocrystals prepared by cation exchange (25 °C, 2 h) and subsequent ion diffusion under 90 °C for different time. **b**, The corresponding intensity change of Tb^{3+} emissions as a function of ion diffusion time. **c**, Upconversion luminescence of the Eu^{3+}-exchanged $NaGdF_4$:Yb/Tm@$NaGdF_4$ nanocrystals prepared by cation exchange (25 °C, 2 h) and subsequent ion diffusion under 90 °C for different time. **d**, The corresponding intensity change of the Eu^{3+} emissions as a function of ion diffusion time. Note that all the spectra are normalized at 450 nm.

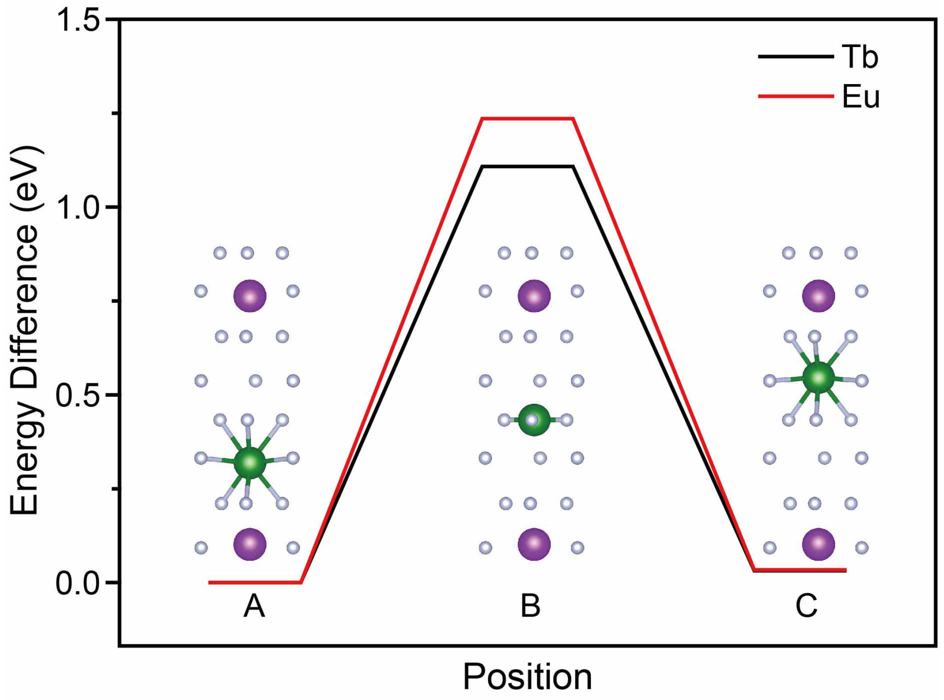

Supplementary Figure 31. Variation of the total energy calculated for a single dopant migrating in the $NaGdF_4$ crystal lattice along (001) surface normal. A, B and C correspond to the initial state, transition state and final state of the doped crystal, respectively. Tb/Eu, Gd, F atoms are represented by green, purple, and white spheres, respectively.

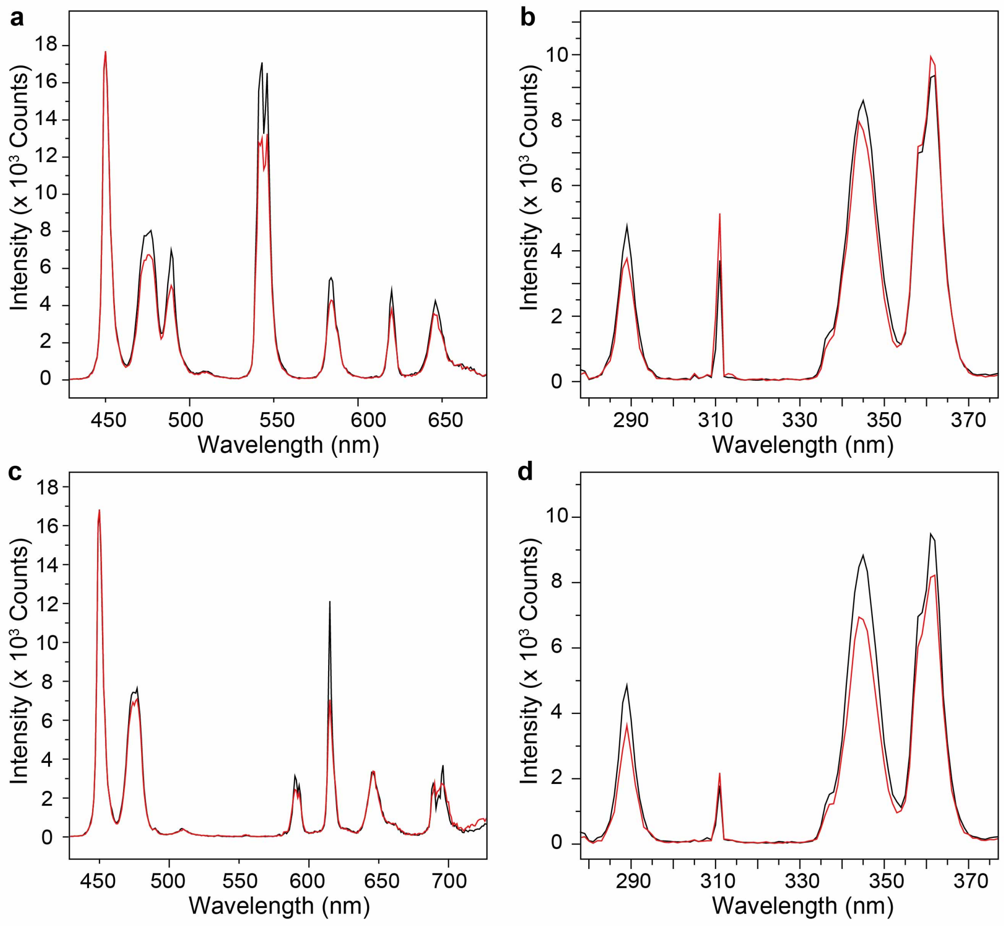

Supplementary Figure 32. Cation exchange and ion diffusion effect on upconversion emission of the exchanged nanocrystals. a-b, Upconversion luminescence of the Tb^{3+}-exchanged $NaGdF_4$:Yb/Tm@$NaGdF_4$ nanocrystals prepared by the (i) and (ii) approach. **c-d**, Upconversion luminescence of the Eu^{3+}-exchanged $NaGdF_4$:Yb/Tm@$NaGdF_4$ nanocrystals prepared by the (i) and (ii) approach. Note that (i) cation exchange at 90 °C for 1 h (black line) and (ii) cation exchange at 25 °C for1 h and ion diffusion at 90 °C for 1h (red line).

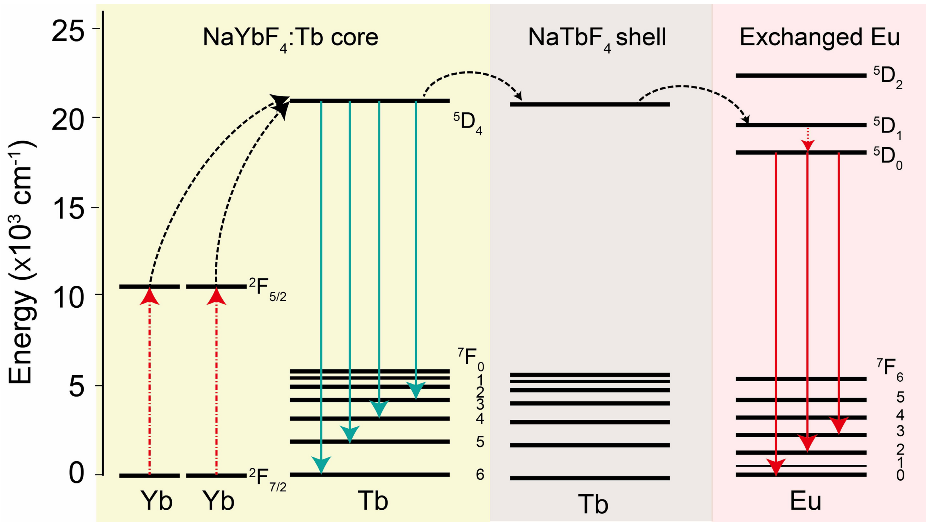

Supplementary Figure 33. Proposed energy transfer mechanism in Eu^{3+}-exchanged $NaYbF_4$:Tb@$NaTbF_4$ core–shell nanocrystals. Note that only partial energy levels of Tb^{3+} and Eu^{3+} are shown for clarity. The dashed/dotted, dashed, dotted, and full arrows represent photon excitation, energy transfer, cross-relaxation, and emission processes, respectively.

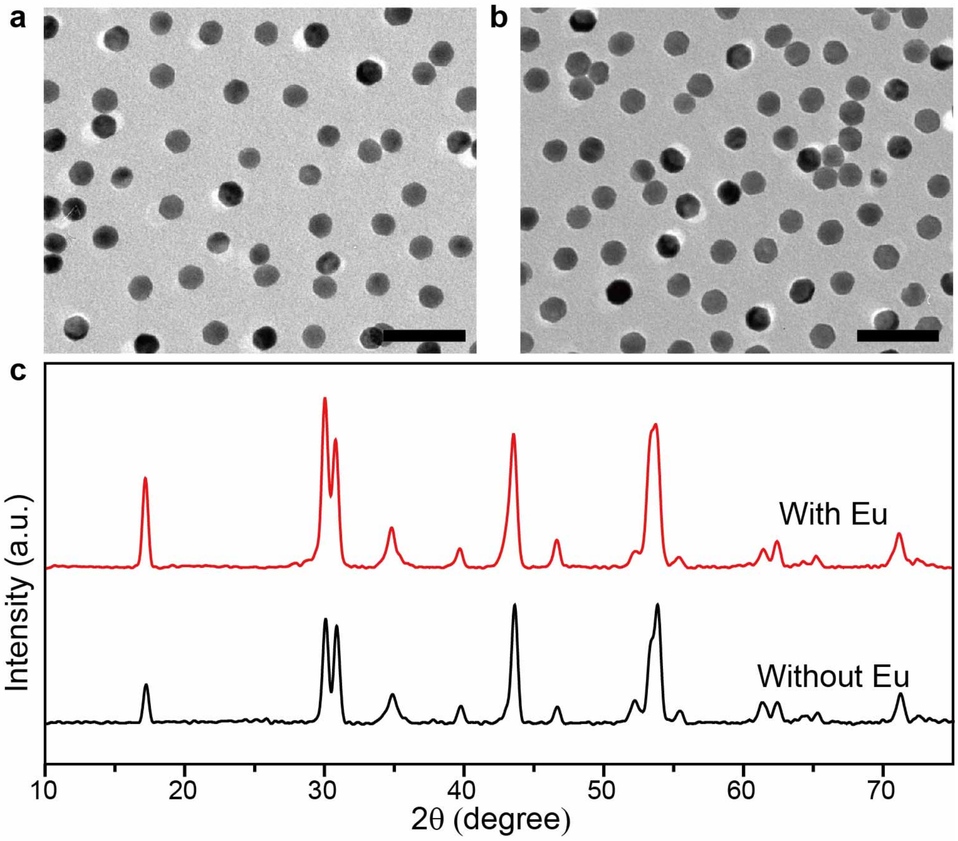

Supplementary Figure 34. Characterizations of the as-prepared Tb^{3+}-activated nanocrystals. a, TEM image of $NaYbF_4$:Tb@$NaTbF_4$ core–shell nanocrystals. **b**, TEM image of $NaYbF_4$:Tb@$NaTbF_4$ nanocrystals obtained after cation exchange with Eu^{3+} ions at 90 °C. Scale bars: 100 nm. **c**, Corresponding x-ray diffraction patterns of the nanocrystals before (black line) and after (red line) cation exchange with Eu^{3+} ions at 90 °C.

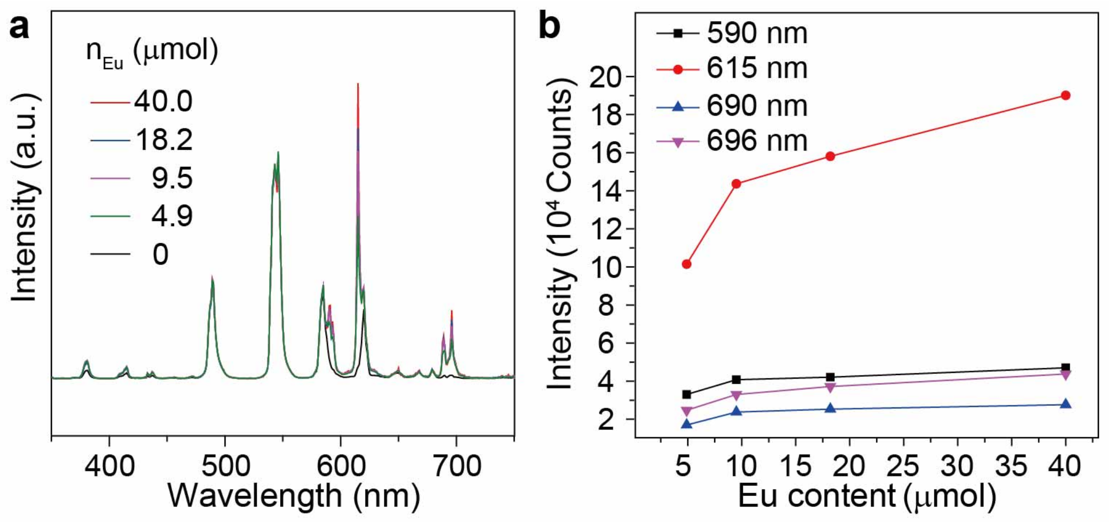

Supplementary Figure 35. Eu^{3+} concentration-dependent emission of the cation-exchanged nanocrystals. a, Upconversion luminescence of the nanocrystals prepared by cation exchange of $NaGdF_4$:Yb/Tm@$NaGdF_4$ nanocrystals (26.2 mg) with varied concentrations of Eu^{3+} at 90 °C. **b**, The corresponding intensity change in Eu^{3+} emission as a function of concentration.

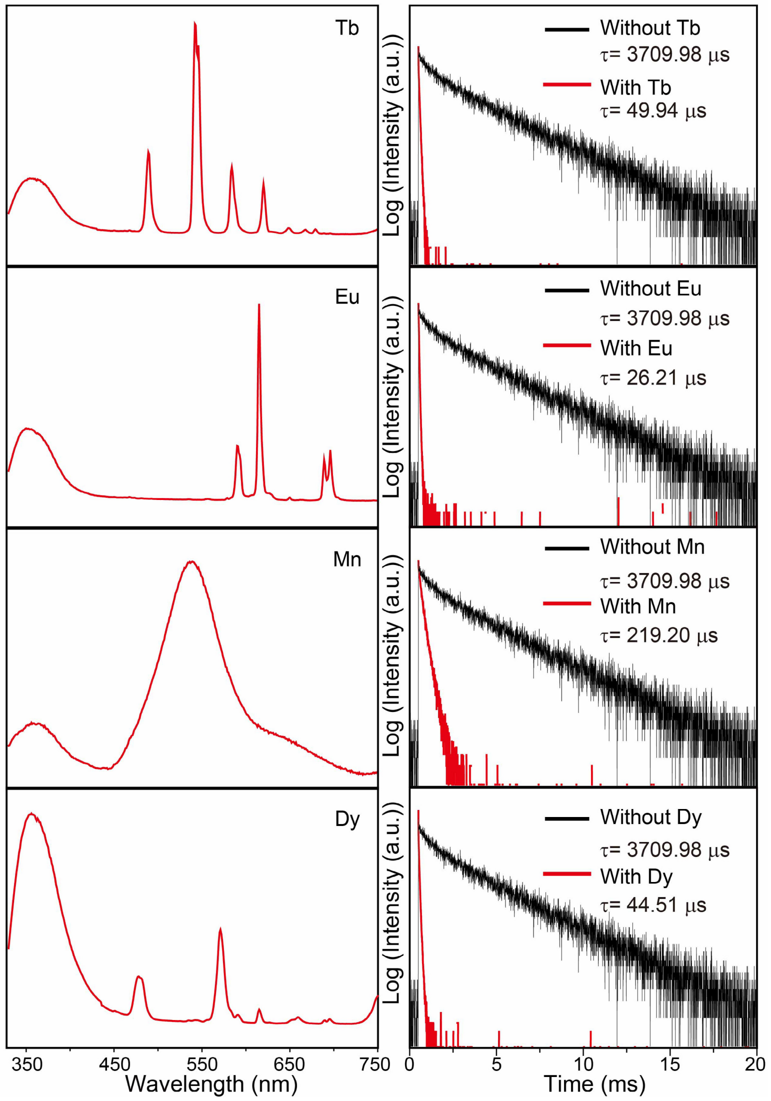

Supplementary Figure 36. Optical Investigations of the cation-exchanged $NaGdF_4$:Ce@$NaGdF_4$ nanocrystals. The down-conversion activator emissions (left panel) and the decay curves of Gd^{3+} emission at 311 nm ($^6P_{7/2} \rightarrow {}^8S_{7/2}$ transition) (right panel) of the nanocrystals (1 mL), prepared by reacting $NaGdF_4$:Ce@$NaGdF_4$ nanocrystals (26.2 mg) with Tb^{3+} (5 μmol), Eu^{3+} (5 μmol), Mn^{2+} (25 μmol), and Dy^{3+} (1 μmol), respectively. The luminescence was recorded under excitation at 254 nm.

Supplementary Tables

Synthetic approach	Sample	Phase	Quantum Yield (%)
**Co-precipitation**	$NaGdF_4$:Yb/Tm (49/1 %)@$NaGdF_4$:Tb (15 %)	Hexagonal	0.86
	$NaGdF_4$:Yb/Tm (49/1 %)@$NaGdF_4$:Eu (15 %)	Hexagonal	0.79
**Cation exchange**	$NaGdF_4$:Yb/Tm (49/1 %)@$NaGdF_4$:Tb	Hexagonal	0.77
	$NaGdF_4$:Yb/Tm (49/1 %)@$NaGdF_4$:Eu	Hexagonal	0.53
	$NaGdF_4$:Yb/Tm (49/1 %)@$NaGdF_4$:Mn	Hexagonal	0.62

Supplementary Table 1. Upconversion quantum yield measurements of different lanthanide-doped materials obtained by co-precipitation and cation exchange approach.

	Formation Energy (eV)	Diffusion Barrier (eV)	Charge Transfer (e)
**Ce**	0.1225	1.204	2.2729
**Eu**	0.1039	1.2353	2.1636
**Tb**	-0.006	1.1079	2.2393
**Dy**	-0.0024	1.1045	2.234
**Mn**	1.8175	1.0381	1.6974

Supplementary Table 2. Formation energies, diffusion energy barriers and charge transfer values calculated for a collection of metal-ion-doped β-$NaGdF_4$ nanocrystals.

Supplementary Notes

Supplementary Note 1: Structural studies of nanocrystals

The $NaGdF_4$:Yb/Tm@$NaGdF_4$ nanocrystals were synthesized as tempate for cation exchange according to a co-precipitation method[1]. A representative TEM image reveals a spherical shape of the nanocrystals with an average size of 21 nm (Supplementary Fig. 1a,b). The X-ray powder diffraction pattern of the as-synthesized sample can be well indexed to a hexagonal-phased $NaGdF_4$ structure (Supplementary Fig.1c).

Oleate ligands have a strong absorption in the UV region, thus resulting in strong quenching of the migration energy stored in Gd sublattice. To overcome this problem, the oleate ligands on the particle's surface were removed by an acid-treatment approach according to the previous report[2]. The complete removal of oleate ligands was further confirmed by FTIR spectroscopic analysis (Supplementary Fig. 2). As shown in Supplementary Fig. 3, the emission intensity of Gd^{3+} at 311 nm was much stronger than that of oleate-capped nanocrystals. The sharp emission at 311 nm is attributed to the radiative decay from $^6P_{7/2}$ state to the ground state of Gd^{3+} ions. In addition, ligand-free $NaGdF_4$:Yb/Tm@$NaGdF_4$ nanocrystals without the oleate ligand stabilization could be well dispersed in an aqueous solution and subsequently amenable for cation exchange.

In our study, the same size, phase, and morphology of the $NaGdF_4$:Yb/Tm(49/1%)@$NaGdF_4$ nanocrystals are found to be largely retained after the cation-exchange treatment. A representative high-resolution STEM image shown in Supplementary Fig. 4 reveals the preservation of the highly crystalline hexagonal structure of nanocrystals after cation exchange with Eu^{3+} ions. Elemental analysis and EELS line scan result carried out at single particle levels provide further evidence of the cation exchange process. Both elemental analyses show a higher Eu^{3+} content at the particle's surface and a higher Gd^{3+} content in the inner layer (Supplementary Fig. 10). Notably, the exchange process did not introduce any detectable surface defects.

We next examined the size and morphology evolution of the Gd^{3+}-based nanocrystals in the presence of Tb^{3+} or Eu^{3+} ions by TEM imaging analysis. In our study, Tb^{3+} or Eu^{3+} ions (15 μmol each) were added to react with ligand-free $NaGdF_4$:Yb/Tm(49/1%)@$NaGdF_4$ nanocrystals (26.2 mg) at room temperature. TEM image comparison of the nanocrystals obtained before and after the cation-exchange process indicated no sign of change in particle size or morphology (Supplementary Fig. 5). To further investigate the temperature effect on the particle's size and morphology, we performed cation-exchange reactions of the ligand-free nanocrystals (26.2 mg) with Tb^{3+} or Eu^{3+} ions (15 μmol each) at temperatures of 25, 50, 70, and 90 °C, respectively. TEM imaging and size

distribution analysis did not show any noticeable changes in size and morphology (Supplementary Figs 6 and 7). The nanocrystals obtained through cation exchange at room temperature and 90 °C could be well indexed to the hexagonal phase (Supplementary Figs 8 and 9).

Supplementary Note 2: Inductively coupled plasma mass spectroscopic (ICP-MS) analysis

To validate that our synthesis is governed by a cation exchange process, we carried out ICP-MS analysis to examine Gd^{3+} content in the solution and Tb^{3+} content in the nanoparticles after treatment of $NaGdF_4$:Yb/Tm@$NaGdF_4$ nanoparticles with $TbCl_3$. As shown in Supplementary Fig. 11, with increasing $TbCl_3$ concentration for cation exchange reaction, the Gd^{3+} content in the solution and Tb^{3+} content in the nanoparticles both significantly increased (Supplementary Fig. 12). The similar trend of ICP-MS test for Gd^{3+} content was observed in the $EuCl_3$-treatment nanoparticle solution (Supplementary Fig. 13). These data demonstrate that our synthesis is governed by the cation exchange process, rather than the deposition of the shell containing Tb^{3+}.

To further confirm our hypothesis, we carried out ICP-MS analysis to examine Gd^{3+} content in the solution after treatment of nanoparticles with Tb^{3+} under different reaction temperatures. We found that the Gd^{3+} content discharged from nanoparticles gradually increased with increasing the reaction temperature (Supplementary Fig. 14), further substantiating the occurrence of cation exchange.

Supplementary Note 3: Optimization of cation exchange-induced color tuning

For luminescence tuning via cation exchange, energy migration through Gd^{3+} sublattices is of significance because it can bridge the energy transfer from the inner layers of the nanoparticle to its surface. To validate this hypothesis, we prepared two sets of $NaYF_4$:Yb/Tm@$NaYF_4$ and $NaGdF_4$:Yb/Tm@$NaGdF_4$ nanoparticles and treated them with Tb^{3+} separately. As a result, no emission of Tb^{3+} could be detected from Y^{3+}-based nanocrystals, while a strong Tb^{3+} emission was observed from Gd^{3+}-based nanocrystals (Supplementary Fig. 15).

In a typical energy migration upconversion (EMU) process involving 980 nm excitation, Yb^{3+} ions harvest the excitation energy and subsequently populate the excited states of Tm^{3+} via multi-step energy transfer upconversion process. The excitation energy stored at the $^1I_6$ state of Tm^{3+} ions is further transferred to the $^6P_{7/2}$ state of Gd^{3+}, followed by energy migration over the host lattice to the particle surface. Ultimately, the excitation energy is trapped by the surface-exchanged activators, allowing the emission color modulation to be achieved. On the basis

of this principle, the emission color could be readily modulated by varying the composition and concentrations of the activator precursors (Supplementary Fig. 16).

The color tuning strategy by cation exchange is strongly dependent on three parameters, namely, the reaction time, the concentration of the exchange ion, and the reaction temperature. To investigate the influence of reaction time on the optical property of the resulting nanocrystals upon cation exchange, we prepared an aqueous solution of ligand-free $NaGdF_4$:Yb/Tm@$NaGdF_4$ nanocrystals (0.9 mL; 26.2 mg). After addition of Tb^{3+} ions (0.2 M; 100 μL) at room temperature, the upconversion luminescence of the colloidal solution was continuously monitored at a time interval of 1 min. By benchmarking the luminescence profile of Tb^{3+} with the Tm^{3+} emission at 646 nm, we noticed that the emission of Tb^{3+} was observed right at the onset of cation addition. Then the emission intensity of Tb^{3+} was gradually boosted, reaching a plateau after 8 min (Supplementary Fig. 19).

To optimize the concentration of the exchanged activators for intense emissions, we performed a series of cation exchange experiments by mixing a fixed quantity of $NaGdF_4$:Yb/Tm@$NaGdF_4$ nanoparticles with Tb^{3+} ions of different concentrations at room temperature for 1 hour. As shown in Supplementary Fig. 17, with increasing Tb^{3+} concentration, the emission intensity of Tb^{3+} initially increased and then gradually decreased owing to the concentration quenching effect, while the intensity of Gd^{3+} emission at 311 nm ($^6P_{7/2} \rightarrow {}^8S_{7/2}$ transition) decreased gradually (Supplementary Fig. 17c). The decay curves of Gd^{3+} emissions from different batches of nanoparticles obtained with increasing Tb^{3+} concentration reveal a consistent, steady decrease in emission lifetime as shown in Supplementary Fig. 17d, providing further evidence of success in the cation exchange process. In reference to Tm^{3+} emission at 450 nm, we found that the optimal Tb^{3+} concentration for maximum emission intensity was estimated to be 15 mM by evaluating the emission profiles of Tb^{3+} at 546 nm (Supplementary Fig. 17b).

We next prepared a set of $NaGdF_4$:Yb/Tm@$NaGdF_4$ nanoparticles surface-exchanged with Eu^{3+} ions at different concentrations under room temperature. Photoluminescence investigations show that the Eu^{3+} emission intensity of the as-synthesized nanoparticles increases up to 0.25 mM of Eu^{3+} concentration and then decreases (Supplementary Fig. 18).

Reaction temperature is another important factor that dictates the cation exchange process. To investigate the temperature effect, we performed a series of reactions between $NaGdF_4$:Yb/Tm@$NaGdF_4$ nanocrystals (26.2 mg/mL) and $TbCl_3$ (15 μmol) or $EuCl_3$ (4 μmol) at different temperatures (25, 50, 70, 90 °C) for 30 min, respectively. As shown in Supplementary Fig. 20, we found that the cation exchange reaction carried out at 90 °C

gives rise to nanoparticles with Tb^{3+} or Eu^{3+} emission much intense than those obtained at lower temperatures, suggesting the active role of the reaction temperature in promoting the cation exchange process.

Supplementary Note 4: Photoluminescence investigation of cation-exchanged nanocrystals

Tb^{3+} and Eu^{3+} exchange. To obtain the optimal emission intensity, we prepared a series of $NaGdF_4$:Yb/Tm@$NaGdF_4$ nanocrystals by cation exchange reaction with different amounts of activators (Tb^{3+} and Eu^{3+}) at 90 °C. Supplementary Figs 21 and 22 show the upconversion luminescence of the as-prepared nanocrystals after the cation exchange reaction at 90 °C. By assessing the upconversion emission of Tb^{3+} or Eu^{3+} and using the Tm^{3+} emission at 646 nm as a reference, the optimal Tb^{3+} and Eu^{3+} concentrations for maximum emission intensity were estimated to be 5 and 4 mM, respectively.

Ce^{3+} exchange. Supplementary Fig. 23a displays upconversion emission spectra of the nanocrystals obtained by the cation exchange reaction between $NaGdF_4$:Yb/Tm@$NaGdF_4$ nanocrystals and Ce^{3+} ions at room temperature. An emission band in the spectral range of 300 to 430 nm could be observed for the colloidal solution after Ce^{3+} exchange, which can be ascribed to the 4f5d → 4f transition of Ce^{3+}. The decrease in the emission lifetime of Gd^{3+} at 311 nm reveals the partial substitution of Gd^{3+} with Ce^{3+} in the host lattice (Supplementary Fig. 23b). To the best of our knowledge, this is the first demonstration of upconverted Ce^{3+} emission from hexagonal-phased nanocrystals. The broadband emission of Ce^{3+} in the UV range should be useful particularly for the promotion of photochemical processes.

To optimize the emission of Ce^{3+}, we further prepared a collection of $NaGdF_4$:Yb/Tm@$NaGdF_4$ nanocrystals treated with Ce^{3+} ions at different concentrations at room temperature. As shown in Supplementary Fig. 24, with increasing Ce^{3+} concentration, the emission intensity of Ce^{3+} increased gradually and then reached a plateau in reference to Tm^{3+} emission at 450 nm.

As shown in Supplementary Fig. 25a, a record-long lifetime of 1.6 ms could be detected for Ce^{3+} emission at 380 nm from the resulting Ce^{3+}-exchanged $NaGdF_4$:Yb/Tm@$NaGdF_4$ nanocrystals. Notably, this lifetime is much longer than that of Tm^{3+} emissions at 346 (221 μs) and 364 nm (290 μs), which allows the emission profile of Ce^{3+} to be separated from the Tm^{3+} emission by time-resolved emission spectroscopy (Supplementary Fig. 25b, c).

Mn^{2+} exchange. Multicolor upconversion luminescence has been achieved by varying relative emission ratios at different wavelengths through control of dopant combination and concentration. Most of these color tuning

strategies are restricted to lanthanide ions. Indeed, the realization of transition metal-activated upconversion luminescence still remains a major challenge. High doping concentration of Mn^{2+} ions often leads to phase transformation during the crystal growth process[3]. To this end, we carried out additional experiments for realizing upconversion from Mn^{2+}-activated nanocrystals by the process of cation exchange. Specifically, we performed the reaction by mixing ligand-free $NaGdF_4$:Yb/Tm@$NaGdF_4$ core-shell nanocrystals and $MnCl_2$ in an aqueous solution at 90 ^{o}C. The resulting nanocrystals give rise to an intense emission in the range of 440 to 660 nm, corresponding to the characteristic $^4T_{1g}(G) \rightarrow {}^6A_{1g}(S)$ transition of Mn^{2+} (Supplementary Fig. 26a and Supplementary Fig. 27). This emission band stems from energy transfer from the excited state ($^6P_{7/2}$) of Gd^{3+} to the $^4T_{1g}(G)$ state of Mn^{2+}, as evidenced by the decreased lifetime in Gd^{3+} emission at 311 nm after cation exchange (Supplementary Fig. 26b). Surprisingly, we observed an ultralong lifetime (~56.7 ms) of Mn^{2+} emission at 550 nm from the Mn^{2+}-exchanged nanocrystals. To the best of our knowledge, this is the longest lifetime ever reported for Mn^{2+}-activated upconversion nanocrystals[4-9].

The measurement of the absolute upconversion quantum yield was carried out by using an optical spectrometer with an integration sphere. The 980-nm excitation light was used to excite the sample. Through total internal reflection in the integrating sphere, the upconversion emission and the excitation light then detected by an extended red photomultiplier (PMT, spectral range 200-1010 nm). Subsequently, the recorded spectra were calibrated. The absolute quantum yield (QY) was determined by QY = P_{sample} / (S_{ref} - S_{sample}), where P_{sample} is the integrated emission intensity of the sample, S_{ref} and S_{sample} are the respective integrated intensities of the excitation light which is not absorbed by the reference or the sample. In our study, aqueous solutions of the ligand-free nanocrystals were used as the sample while the ultrapure water was used as the reference. The quantum yields of the samples obtained by the process of cation exchange are comparable to those obtained by the conventional co-precipitation approach (Supplementary Table 1).

Supplementary Note 5: Mechanistic study of cation exchange-induced color tuning

The cation exchange reaction is strongly influenced by the concentration of the exchange ions or the reaction temperature. To validate this effect, we carried out inductively coupled plasma mass spectroscopy (ICP-MS) analysis of the supernatant of the cation exchanged colloidal solution after dialysis. As shown in Supplementary Fig. 28a, with increasing amounts of Tb^{3+} at room temperature (25 ^{o}C), the Gd content discharged from the

nanocrystals gradually increases after the treatment of $NaGdF_4$:Yb/Tm@$NaGdF_4$ nanocrystals with Tb^{3+}. That suggests that high concentrations of exchanged ions could promote the process of cation exchange. Next, we performed a control reaction between $NaGdF_4$:Yb/Tm@$NaGdF_4$ nanocrystals and Tb^{3+} ions at 90 °C. After dialysis, the ICP-MS result revealed that the Gd^{3+} content discharged from the nanocrystals is higher as compared with that attainable at 25 °C. This clearly demonstrates that the cation exchange reaction could occur more easily at an elevated temperature.

Supplementary Fig. 28b shows that the cation exchange reaction through the use of Mn^{2+} is less favorable than that driven by lanthanide ions, as evidenced by the relatively small amount of Gd^{3+} ions obtained from the supernatant of the colloidal solution after the reaction. This can be explained by the fact that the calculated formation energy in Mn-doped nanocrystals is higher and the transferred charge between Mn and Gd is lower (Supplementary Table 2 and Supplementary Fig. 29).

On the basis of optical investigations, we found that the nanoparticles obtained by cation exchange reactions under higher temperature tend to afford intense activator emission (Supplementary Fig. 20). We attributed this effect to the increased cation exchange rate and subsequently accelerated ion diffusion from the particle's outmost surface to its inner layer under the influence of elevated temperatures. Under a high temperature, the increased cation exchange rate can promote more activators to exchange with the nanocrystals. In addition, the subsequent step of ion diffusion can considerably facilitate the exchanging process at a greater depth. Consequently, the concentration and surface quenching can be largely mitigated, thus resulting in an enhanced emission. To validate the occurrence of ion diffusion, we first performed cation exchange reactions by mixing $NaGdF_4$:Yb/Tm@$NaGdF_4$ nanocrystals with Tb^{3+} or Eu^{3+} activators at room temperature for 1 h, at which time the resultant nanocrystals were isolated. Subsequently, we heated the nanocrystals to 90 °C. By monitoring the emission intensity of the activators in reference to Tm^{3+} emission at 450 nm, we observed that the intensity increases gradually as a function of heating time and reaches a plateau (Supplementary Fig. 30). The enhanced emission is attributed to the accelerated rate of ion diffusion at elevated temperatures, as revealed by our calculated moderate diffusion barriers (Supplementary Table 2 and Supplementary Fig. 31).

The increased cation exchange rate and ion diffusion effects under high temperature treatment both contribute to enhanced activator emissions. To further probe the two effects on the optical property of the cation-exchanged nanoparticles, two comparative experiments were carried out. For the first experiment, we mixed the activator

precursors with $NaGdF_4$:Yb/Tm@$NaGdF_4$ nanocrystals at 90 °C for 1 h. For the other experiment, we first mixed the nanocrystals with the activator precursors at room temperature for 1 h. After isolation of the resulting cation-exchanged nanocrystals from the solution, we then heated the nanocrystals at 90 °C for 1h. Such synthetic process ensures that the cation exchange event occurs at room temperature while the ion diffusion proceeds at 90 °C. As anticipated, we recorded a much stronger emission of the activators and a large decrease of Gd^{3+} emission in the resulting product prepared by the cation exchange reaction at 90 °C (Supplementary Fig. 32). Taken together, these data further confirm that the enhanced emission intensity is attributed to the improved processes of cation exchange and ion diffusion at high temperatures.

Supplementary Note 6: Tb^{3+}-mediated energy migration upconversion

To demonstrate the generality of our approach for color tuning in other systems, we carried out cation exchange reactions in Tb^{3+}-activated nanoparticles. As a proof-of-concept experiment, we prepared the ligand-free $NaYbF_4$:Tb(30%)@$NaTbF_4$ core-shell nanocrystals and then performed the cation exchange reaction with Eu^{3+} ions in water at 90 °C. Following the 980 nm excitation, the excitation energy is firstly harvested by Yb^{3+} and transferred to neighboring Tb^{3+} ions via a cooperative sensitization upconversion process. After that, Tb^{3+} is expected to transfer its energy to exchanged Eu^{3+} ions, thereby allowing the particle's emission to be modulated (Supplementary Fig. 33).

Prior to luminescence investigations, we examined the size, phase and morphology of the as-prepared nanocrystals before and after the treatment of Eu^{3+} ions at 90 °C. As displayed in Supplementary Fig. 34, it is evident that the cation exchange process did not induce noticeable changes in particle size, phase, and morphology. To validate the emission tunability enabled by our strategy, we performed cation exchange reactions by mixing $NaYbF_4$:Tb@$NaTbF_4$ nanocrystals (26.2 mg) with Eu^{3+} at varying concentrations. As shown in Supplementary Fig. 35, the Eu^{3+} emission increases gradually with increasing Eu^{3+} concentration.

Supplementary Note 7: Down-conversion luminescence

Our cation exchange approach is also applicable to fine-tuning of down-conversion luminescence. To validate this hypothesis, we reacted $NaGdF_4$:Ce(15%)@$NaGdF_4$ core-shell nanoparticles with different types of activator (Tb^{3+}, Eu^{3+}, Dy^{3+}, and Mn^{2+}) at a temperature of 90 °C. As shown in Supplementary Fig. 36, intense emissions from the purified nanoparticles after the treatment with Tb^{3+}, Eu^{3+}, Dy^{3+}, and Mn^{2+} ions, respectively, can be clearly observed under 254-nm excitation. These emissions are attributed to the energy transfer from Gd^{3+} to the

exchanged activators, as evidenced by the decreased emission lifetime of Gd^{3+} at 311 nm.

Supplementary Methods

Materials: gadolinium(III) acetate hydrate (99.9%), ytterbium(III) acetate hydrate (99.9%), thulium acetate hydrate (99.9%), terbium(III) chloride hexahydrate (99.9%), europium(III) chloride hexahydrate (99.9%), sodium hydroxide (NaOH; >98%), ammonium fluoride (NH_4F; >98%), 1-octadecene (90%), and oleic acid (90%) were all purchased from Sigma-Aldrich. Unless otherwise noted, all the chemicals were used without further purification.

Synthesis of $NaGdF_4$:Yb/Tm (49/1 %) core nanoparticles: In a typical procedure[10], an aqueous solution (2 mL) of $Ln(CH_3CO_2)_3$ (0.4 mmol, Ln = Gd, Yb, and Tm) was mixed with oleic acid (4 mL) in a 50 mL flask. The mixture was heated at 150 °C under stirring. After 30 min, octadecene (6 mL) was added to the mixture and kept at 150 °C for another 30 min. After cooling down to 50 °C, a methanol solution containing NH_4F (1.36 mmol) and NaOH (1 mmol) was added and stirred for 2 h. After the removal of the low boiling solvent, the mixture was heated to 290 °C and then kept for 1.5 h under a flow of argon. The resulting nanoparticles were collected by addition of ethanol, washed with cyclohexane and ethanol several times, and re-dispersed in cyclohexane.

Synthesis of $NaGdF_4$:Yb/Tm@$NaGdF_4$ core-shell nanoparticles. In a typical experiment, an aqueous solution (2 mL) of 0.4 mmol of $Gd(CH_3CO_2)_3$ was added to a 50 mL flask containing oleic acid (4 mL). The mixture was then heated at 150 °C for 30 min under stirring, followed by addition of 1-octadecene (6 mL). After another 30 min, the mixture was cooled down to 50 °C. The as-synthesized $NaGdF_4$ core nanoparticles in 4 mL of cyclohexane were added along with a methanol solution of NH_4F (1.36 mmol) and NaOH (1 mmol). After 30 min, the reaction temperature was increased to 100 °C to remove the low boiling point solvents. Then the solution was heated at 290 °C under an argon flow for 1.5 h before being cooled to room temperature. The resulting nanoparticles were washed with ethanol and redispersed in cyclohexane.

Synthesis of $NaYbF_4$:Tb (30%) core nanoparticles. In a typical experiment, an aqueous solution (2 mL) of 0.4 mmol of $Yb(CH_3CO_2)_3$ and $Tb(CH_3CO_2)_3$ was added to a 50 mL flask charged with oleic acid (3 mL) and 1-octadecene (7 mL). The resulting mixture was heated at 150 °C for 1 h and then cooled down to 50 °C. Subsequently, a methanol solution (6 mL) containing NaOH (1 mmol) and NH_4F (1.6 mmol) was added. After 0.5 h, the methanol was removed. Then the mixture was heated at 290 °C under an argon flow for 1.5 h. After cooling

down to room temperature, the resulting $NaYbF_4$:Tb core nanoparticles were collected by centrifugation, washed with ethanol, and finally dispersed in cyclohexane.

Synthesis of $NaYbF_4$:Tb@$NaTbF_4$ core-shell nanoparticles. The $NaYbF_4$:Tb@$NaTbF_4$ core-shell nanoparticles were prepared by an epitaxial growth on the as-synthesized $NaYbF_4$:Tb (30 %) core nanoparticles. In a typical procedure, an aqueous solution (2 mL) of $Tb(CH_3CO_2)_3$ (0.4 mmol) was mixed with oleic acid (3 mL) and 1-octadecene (7 mL). The mixture was then heated at 150 °C for 1 h to form Tb-oleate complexes. After cooling down to 50 °C, the $NaYbF_4$:Tb core nanoparticles were added, along with a methanol solution of NH_4F (1.6 mmol) and NaOH (1 mmol). After the reaction for 0.5 h, the methanol was removed and the mixture was then heated at 290 °C under an argon flow for 1.5 h before cooling down to room temperature. The resulting $NaYbF_4$:Tb@$NaTbF_4$ core-shell nanoparticles were collected by centrifugation, washed with ethanol, and dispersed in cyclohexane.

Synthesis of $NaGdF_4$:Ce (15 %) core nanoparticles: In a typical procedure, an aqueous solution (2 mL) of $Gd(CH_3CO_2)_3$ (0.34 mmol) and $Ce(CH_3CO_2)_3$ (0.06 mmol) was mixed with oleic acid (4 mL) in a 50 mL flask. The mixture was then heated to 150 °C under stirring. After heating for 30 min, octadecene (6 mL) was added to the mixture and stirred at 150 °C for another 30 min. After cooling down to 50 °C, a methanol solution containing NH_4F (1.28 mmol) and NaOH (1 mmol) was added and stirred for 2 h. Subsequently, the low boiling solvent was removed *in vacuo*. The resultant residue was heated to 290 °C and kept at this temperature for 1.5 h under the protection of argon. The resulting nanoparticles were collected by addition of ethanol, washed with cyclohexane and ethanol several times, and re-dispersed in cyclohexane.

Synthesis of $NaGdF_4$:Ce@$NaGdF_4$ core-shell nanoparticles. In a typical experiment, to a 50 mL flask charged with oleic acid (4 mL) was added an aqueous solution (2 mL) of 0.4 mmol of $Gd(CH_3CO_2)_3$. The mixture was then heated at 150 °C for 30 min under stirring, followed by addition of 1-octadecene (6 mL). After another 30 min of reaction, the mixture was cooled down to 50 °C. The as-synthesized $NaGdF_4$ core nanoparticles in 4 mL of cyclohexane were added alongside a methanol solution of NH_4F (1.28 mmol) and NaOH (1 mmol). After 30 min, the reaction temperature was increased to 100 °C to remove the low boiling point solvents *in vacuo*. The residue was heated at 290 °C under an argon flow for 1.5 h and then cooled to room temperature. The resulting nanoparticles were collected by addition of ethanol, washed with cyclohexane and ethanol, and redispersed in cyclohexane.

Synthesis of ligand-free nanoparticles. The as-prepared oleic acid-capped nanoparticles were dispersed in a mixed solution of ethanol (1mL) and HCl (0.2 M; 1 mL)[2]. The mixture was sonicated for 5 min and collected by centrifugation. Subsequently, the resulting products were washed with ethanol/H_2O several times and re-dispersed in H_2O.

Cation exchange process. In a typical experiment, a stock solution (1 mL) of the as-prepared ligand-free core-shell nanoparticles (e.g. $NaGdF_4$:Yb/Tm@$NaGdF_4$, $NaYbF_4$:Tb@$NaTbF_4$, and $NaGdF_4$:Ce@$NaGdF_4$) was mixed with an aqueous solution of $LnCl_3$ (Ln = Tb^{3+}, Eu^{3+}, Dy^{3+}, or Ce^{3+}) or $MnCl_2$. The resulting mixture was shaken thoroughly and heated at room temperature (especially for Ce^{3+}) or 90 °C for 1h. Subsequently, the products were collected by centrifugation, washed with H_2O several times, and re-dispersed in H_2O.

Synthesis of nanoparticle-modified polystyrene microspheres: In a typical experiment, an aqueous solution (100 μL) of the ligand-free nanoparticles (~20 mg) was mixed with 200 μL of polystyrene butanol solution. The mixture was incubated at room temperature for 30 min. After that, the nanoparticle-modified microspheres were obtained by centrifugation at 6000 rpm for 5 min, followed by washing with butanol and H_2O several times, and finally dispersed in H_2O.

Computational details: In order to unravel the cation exchange barrier between the host Gd^{3+} and guest ions (Ce^{3+}, Eu^{3+}, Tb^{3+}, Dy^{3+} or Mn^{2+}), we calculated the formation energies of Ce-, Eu-, Tb-, Dy- and Mn-doped β-$NaGdF_4$ nanocrystals and the corresponding diffusion barriers of these guest atoms in the host lattices using first principles calculations based on the density functional theory (DFT). All calculations were carried out using the Vienna *ab initio* simulation package (VASP) with generalized gradient approximation (GGA) and the projector-augmented wave (PAW) method[11-14]. A kinetic energy cut-off of 500 eV was used for the plane wave. The formation energy of the doped system is given by

$$E_{\text{form}}\left(\text{Na}_N\text{R}_m\text{Gd}_{N\text{-}m}\text{F}_{4N}\right) = E\left(\text{Na}_N\text{R}_m\text{Gd}_{N\text{-}m}\text{F}_{4N}\right) - mE\left(\text{NaRF}_4\right) - \left(N-m\right)E\left(\text{NaGdF}_4\right) \quad (1)$$

where *N* is the number of Gd atoms in the system, *m* is the number of the dopant in the system, and R indicates Ce, Eu, Tb, Dy and Mn atoms. Note that *E* denotes to the energy of different systems shown in the parentheses. To determine the lowest energy trajectory for dopant motion, we moved the dopant atom toward the Gd vacancy along the (0 0 1) surface normal in 0.9 Å steps. The lateral position of all atoms is allowed to relax until the forces acting on each atom are smaller than 0.02 eV/Å.

Characterization. Elemental analysis was performed on an Elementar Vario MICRO elemental analyzer. The lifetime and time-resolved emission spectra were measured using an Edinburgh FLSP920 fluorescence spectrophotometer equipped with a xenon arc lamp (Xe900), a microsecond flash-lamp (μF900) and a 980 nm diode laser. The lifetimes (τ) of the luminescence were obtained by fitting the decay curve with a multi-exponential decay function of

$$I(t) = \sum_i A_i e^{-\frac{t}{\tau_i}} \qquad (2)$$

where A_i and τ_i represent the amplitude and lifetime of individual components for multi-exponential decay profiles, respectively. The photos and supporting videos were recorded by a Nikon D90 camera and iPhone 6, respectively.

Scanning transmission electron microscopy measurement: Atomic resolution scanning transmission electron microscopy (STEM), and electron energy loss spectroscopy (EELS) experiments were carried out on an FEI aberration-corrected Titan Cubed S-Twin transmission electron microscope operated at 200 kV. The STEM measurement with a spatial resolution of ~1.0 Å was performed at a 2-μs/pixel scanning rate with 70 μm C2 aperture, spot size 9, a high-angle annular dark-filed (HAADF) detector, and 146 mm camera length. For EELS point analysis, 150 μm C2 aperture, spot size 6, 29.6 mm camera length, 5 mm entrance aperture (collection angle β = 54 mrad) and 1s collection time were used. The EELS line scan was conducted using 70 μm C2 aperture, spot size 9, 29.6 mm camera length, 5 mm entrance aperture (collection angle β = 54 mrad) and 0.1 s/pixel collection time.

Supplementary References

[1] Wang, F., Deng, R. & Liu, X. Preparation of core-shell $NaGdF_4$ nanoparticles doped with luminescent lanthanide ions to be used as upconversion-based probes. *Nat. Protoc.* **9**, 1634–1644 (2014).

[2] Bogdan, N., Vetrone, F., Ozin, G. A. & Capobianco, J. A. Synthesis of ligand-free colloidally stable water dispersible brightly luminescent lanthanide-doped upconverting nanoparticles. *Nano Lett.* **11**, 835–840 (2011).

[3] Tian, G. *et al.* Mn^{2+} Dopant-controlled synthesis of $NaYF_4$:Yb/Er upconversion nanoparticles for in vivo imaging and drug delivery. *Adv. Mater.* **24**, 1226–1231(2012).

[4] Li, X. *et al.* Energy migration upconversion in manganese(II)-doped nanoparticles. *Angew. Chem. Int. Ed.* **127**, 13510–13515 (2015).

[5] Song, E. *et al.* Temperature-tunable upconversion luminescence of perovskite nanocrystals $KZnF_3$:Yb^{3+},Mn^{2+}. *J. Mater. Chem. C* **1**, 4209-4215 (2013).

[6] Reinhard, C., Gerner, P., Valiente, R., Wenger O. S. & Güdel, H. U. Upconversion phenomena in the Yb^{3+} doped transition metal compounds Rb_2MnCl_4 and $CsMnBr_3$. *J. Lumin.* **94–95**, 331-335 (2001).

[7] Gerner, P., Wenger, O. S., Valiente, R. & Güdel, H. U. Green and red light emission by upconversion from the near-IR in Yb^{3+} doped $CsMnBr_3$. *Inorg. Chem.* **40**, 4534-4542 (2001).

[8] Martín-Rodríguez, R., Valiente, R. & Bettinelli, M. Room-temperature green upconversion luminescence in $LaMgAl_{11}O_{19}$:Mn^{2+}, Yb^{3+} upon infrared excitation. *Appl. Phys. Lett.* **95**, 091913 (2009).

[9] Valiente, R., Wenger, O. S. & Güdel, H. U. Near-infrared-to-visible photon upconversion process induced by exchange interactions in Yb^{3+}-doped $RbMnCl_3$. *Phys. Rev. B* **63**, 165102 (2001).

[10] Wang, F. et al. Simultaneous phase and size control of upconversion nanocrystals through lanthanide doping. *Nature* **463**, 1061–1065 (2010).

[11] Kresse, G. & Hafner, J. *Ab initio* molecular dynamics for liquid metals. *Phys. Rev. B* **47**, 558 (1993).

[12] Kresse, G. & Furthmuller, J. Efficiency of ab-initio total energy calculations for metals and semiconductors using a plane-wave basis set. *Comput. Mater. Sci.* **6**, 15-50 (1996).

[13] Kresse, G. & Furthmuller, J. Efficient iterative schemes for ab initio total-energy calculations using a plane-wave basis set. *Phys. Rev. B* **54**, 11169-11186 (1996).

[14] Blochl, P. E. Projector augmented-wave method. *Phys. Rev. B* **50**, 17953-17979 (1994).
